# Transcriptomic Analyses of Normal Human Pancreata Reveal the Presence of Cancer Subtypes that Correlate with Acinar Ductal Metaplasia and Donor Ancestry

**DOI:** 10.1158/2767-9764.CRC-25-0411

**Published:** 2026-01-21

**Authors:** Corey M. Perkins, Jinmai Jiang, Kalyanee Shirlekar, Zachary Greenberg, Md Abu Talha Siddique, Jason Brant, Kiley Graim, Mei He, Sarah Kim, Diana J. Wilkie, Bo Han, Jamel Ali, Pascal Belleau, Astrid Deschênes, Alexander Krasnitz, Mazhar Kanak, Thomas D. Schmittgen

**Affiliations:** 1 Department of Pharmaceutics, College of Pharmacy, University of Florida, Gainesville, Florida.; 2 Florida-California Cancer Research Education and Engagement Health Equity Center.; 3 Department of Biostatistics, University of Florida, Gainesville, Florida.; 4 Department of Computer & Information Science & Engineering, Herbert Wertheim College of Engineering, University of Florida, Gainesville, Florida.; 5 Department of Behavioral Nursing Science, College of Nursing, University of Florida, Gainesville, Florida.; 6 Department of Surgery, University of Southern California, Los Angeles, California.; 7 Department of Chemical and Biomedical Engineering, FAMU-FSU College of Engineering, Tallahassee, Florida.; 8 Simons Center for Quantitative Biology, Cold Spring Harbor Laboratory, Cold Spring Harbor, New York.; 9 Cancer Center, Cold Spring Harbor Laboratory, Cold Spring Harbor, New York.; 10 Department of Transplant Surgery, Virginia Commonwealth University, Richmond, Virginia.

## Abstract

Molecular subtypes exist in pancreatic ductal adenocarcinoma (PDAC); however, comparable subtypes are not known to exist in normal pancreas. A large, racially diverse cohort of normal, human pancreatic acinar cells was obtained from islet transplantation centers. RNA isolated from uncultured acini and those acini that had undergone acinar ductal metaplasia (ADM) was sequenced. Data analysis from 69 uncultured, normal acini showed two clusters of samples, one that aligns with exocrine-resembling tissue (ERT) signatures and another with the classical and basal (C/B) subtypes. Gene expression associated with the ERT signature decreased following 6 days of ADM transdifferentiation, whereas those assigned to the C/B subtype increased. Subtype classification findings were confirmed in two independent cohorts of transcriptomic data of normal pancreas. A direct correlation exists between ancestral admixture and the molecular index of ADM; those specimens with higher African ancestral admixture showed a greater propensity for ADM compared with those with a higher Ameridigenous admixture. The extent of morphologic ADM decreased in the order of Black > White > Hispanic race. Analysis of associated metadata revealed that potential confounders such as diabetes, obesity, age, gender, or specimen handling and processing variables did not influence the distribution of samples across subtype classification. We report that normal human pancreatic acinar tissues group into molecular subtypes like those used to classify PDAC and that subtype assignment and ADM are influenced by the donor’s race.

**Significance:**

Human tissue models provide direct insights into human pancreatic biology and preserve the donor-to-donor heterogeneity present in the general population. By utilizing these models, our study suggests that a subset of normal pancreata exhibits a preexisting permissive state that renders acinar cells more susceptible to early reprogramming and ADM.

## Introduction

Pancreatic ductal adenocarcinoma (PDAC) is one of the well-characterized cancers for multistep progression models that have “Big 4” mutations (*KRAS*, *SMAD4*, *TP53*, and *CDKN2A*; ref. 1). Phenotypic plasticity is a hallmark of cancer (2), and in the pancreas, metaplastic changes exemplify this adaptability, ultimately contributing to tumorigenesis. Studies in mice show that PDAC can originate from either the acinar (3–5) or ductal (6, 7) cells of the pancreas. A well-established pathway for PDAC development begins with acinar cells undergoing acinar ductal metaplasia (ADM), followed by the acquisition of oncogenic *KRAS* mutations and loss of tumor suppressor function (8). Although mechanistic studies on the origins of PDAC using genetically homogeneous, transgenic mouse models have yielded immense knowledge (9), these models lack the intersubject heterogeneity observed in humans. Organoids derived from sorted normal human acinar or ductal cells were transformed following the introduction of *KRAS*^G12D^ and knockout of *CDKN2A*, *TP53*, and *SMAD4*, demonstrating that human PDAC may be derived from either cellular compartment of human pancreas (10).

ADM is a transient, protective mechanism that occurs when pancreatic acinar cells transdifferentiate into cells with a ductal-like phenotype during periods of pancreatic damage (11–13). Dedifferentiation of acinar cells to ductal epithelial cells with embryonic progenitor properties reduces zymogen-associated damage and produces cells with increased proliferative activity, believed to contribute to the regeneration of acinar structures and repopulation of the pancreas (8). ADM is accompanied by a reduction in digestive enzymes (e.g., *AMY2A* and *CPA1*) and increased expression of genes that define normal ductal cells (e.g., *KRT19* and *SOX9*; refs. 5, 14). In the presence of mutant *Kras*, ADM is believed to be irreversible; however, we have recently shown that ADM may be reversed in mice harboring *Kras*^G12D^ using small-molecule epigenetic modulators *ex vivo* (15, 16).

Black patients display an overall significantly greater age-adjusted incidence and mortality from PDAC compared with White patients (17, 18). A recent study showed an improvement in the 5-year survival rate for PDAC between 2015 to 2019 and 2003 to 2006; however, Black patients fared worse than Whites and Hispanics (19). Recognized risk factors for PDAC, including smoking, diabetes, obesity, and chronic pancreatitis, do not completely account for the increased incidence and mortality of PDAC in Black individuals (20). Collectively, Black patients present with PDAC at a younger age, comprise a socioeconomically disadvantaged population, demonstrate more aggressive disease at the time of diagnosis, and have a poorer response following neoadjuvant therapy (20, 21). Although access to care and socioeconomic/environmental factors likely contribute to the observed differences in PDAC with respect to race, alternative contributions are likely. A biological explanation for the higher incidence and mortality of PDAC among Black individuals is possible; however, there is currently insufficient evidence to substantiate this hypothesis.

The transcriptomes of human PDAC have been categorized and classified into unique molecular subtypes, including exocrine, classical, and basal (22–26). The exocrine subtype retains the expression of digestive enzymes and other genes unique to acinar cells and is widely believed to not represent a true molecular PDAC subtype but rather reflects contamination by residual normal acinar tissue within the tumor sample. Classical tumors are enriched for epithelial and pancreatic lineage transcriptomic programs, whereas basal tumors are enriched for TGFβ signaling, WNT signaling, epithelial–mesenchymal transition, and cell-cycle progression (26). Patients with the basal subtype of PDAC have significantly poorer outcomes compared with those with the classical subtype (23, 26). In transgenic mice, PDAC arising from the acinar compartment will develop into the classical subtype, whereas tumors originating from ductal cells are more likely to adopt a basal-like phenotype (27). To our knowledge, the existence of molecular subtypes in normal, human pancreas has not been reported. The human pancreas is notoriously challenging for molecular analysis because of the rapid autodigestion of pancreatic RNA and proteins that occurs shortly after death. In an extensive study of normal pancreases obtained using a donation after brain death protocol, Carpenter and colleagues (28), performed single-cell RNA sequencing (RNA-seq) and spatial transcriptomics on tissues collected from 13 disease-free pancreases and reported that neoplastic pathways in otherwise normal pancreas may be initiated earlier in tumorigenesis than previously understood.

We present a study utilizing normal human acinar tissues collected from deceased organ donors obtained from islet transplantation centers over a span of 7 years. Intersubject heterogeneity of 69 normal pancreatic acinar tissues was maintained. We address the contribution of genetic ancestry on the transcriptomics of normal acinar tissues, as well as the degree to which they undergo ADM. Moreover, we report that normal human pancreatic acinar tissues group into molecular subtypes like those used to classify PDAC.

## Materials and Methods

### Human pancreatic acinar cells

Primary, normal, human acinar cells from deceased organ donors were received from pancreatic islet transplantation centers, Prodo Laboratories, Network for Pancreatic Organ Donors with Diabetes, University of Miami, and Virginia Commonwealth University. Donors remained on life support until the surgery to remove their pancreas. Islet cells were purified from the total pancreatic cell digest by a density gradient purification process using a COBE 2991 Cell Processor. Human acinar cells (1.100–1.115 g/mL) have a higher density than human islets (1.075–1.100 g/mL). Following density gradient centrifugation, islets were collected in earlier fractions, and acinar cells were collected in the later fractions. The cells collected in the last fraction (>99% acinar cells and >95% viability) were shipped on blue ice packs in PIM-T media (1% PIM-G, 2.5% AB serum, 10 μg/mL ciprofloxacin hydrochloride, and 100 μg/mL trypsin inhibitor from *Glycine max*) by overnight carrier. Cell viability was not rechecked upon receipt of the acinar cells in the research laboratory. All 69 samples were wild type for *KRAS* codon 12 as determined from the RNA-seq data (described below). Donor demographics of the 69 organ donors are provided in Supplementary Table S1. Self-reported race of the donors is listed as Black, White, and Hispanic, whereas ancestral admixture is defined as African (AFR), European (EUR), or Ameridigenous (AMR). The study protocol was reviewed and approved by the University of Florida Institutional Review Board (IRB201902530).

### Acinar cell culture

Acinar cells were cultured in two dimensions using growth factor–reduced Matrigel (Corning) in serum-free conditions as previously described (14) and detailed in Supplementary Methods. For the calculation of % ADM, microscopic adhesive grids (Sigma-Aldrich) were adhered to the bottom of each well of a 96-well plate. The number of ductal and acinar cell clusters was microscopically counted at 4× magnification using a brightfield inverted microscope. Acinar cells were identified as small, irregular clusters of cells, whereas ductal cells organized into small spheroid structures characterized by a ring of cells encircling a hollow lumen. Cells that were viewable within 25 individual squares of the adhesive grid were counted (typically 200–300 total cells). The % ADM was calculated as the number of ductal cells divided by the total cellular objects × 100%. Typically, one well of cells was counted at each time point during the ADM process.

### RNA isolation

Matrigel was removed from the cells by a cold centrifugation step as previously described (29). The cell pellets were lysed in TRIzol reagent, and total RNA was isolated using the Qiagen miRNeasy Mini Kit. RNA integrity was assessed, and RNA samples with an RNA integrity number (RIN) greater than 5 were used for cDNA and next-generation sequencing library preparation (Supplementary Table S1).

### Library preparation for Illumina NovaSeq sequencing

RNA-seq libraries were prepared from 60 ng of total RNA using the NEBNext Ultra II Directional RNA Library Prep Kit for Illumina as recommended by the manufacturer. mRNA was first enriched from total RNA using NEBNext Poly(A) mRNA Magnetic Isolation Module. Library size and mass assessed using the Agilent D1000 TapeStation were pooled equimolarly and cleaned with AMPure beads (Omega Bio-Tek). The pooled library was sequenced on the Illumina NovaSeq series platform to a target depth of ∼50 million reads per sample using a 10B flow cell.

### RNA sequence reads

Paired-end sequencing was performed using Illumina NovaSeq series at 2 × 150 cycle sequencing for 69 samples harvested on day 0 in four batches. RNA-seq data processing was performed using the nf-core RNA-seq pipeline v3.12.0 (https://github.com/nf-core/rnaseq). Raw sequencing reads were trimmed using Trim Galore v0.6.7, and reads shorter than 20 bp after trimming were discarded. Read alignment was performed with STAR v2.7.10a to the reference genome GRCh37, and gene expression quantification was done using RSEM v1.3.1. Raw counts were corrected using ComBat-Seq from the sva package v3.52.0 (30) to remove batch-induced technical variation. Filtering of raw counts was performed using edgeR’s v4.2.2 (31), requiring a minimum of 10 counts in at least 70% of samples. The batch-corrected counts were normalized using the trimmed mean of M-values method. Spectral clustering analysis using Spectrum v1.1 (32) was performed on the batch-corrected filtered counts to identify underlying patterns in the data. This analysis revealed two distinct clusters within the log counts per million. All curated datasets were posted to the Gene Expression Omnibus (GEO) repository under accession number GSE295071.

### Continental genetic ancestry

Continental genetic ancestry was inferred from the RNA-seq data as described (33). Each donor was assigned one of AFR, AMR, East Asian, EUR, or South Asian continental ancestries. A detailed protocol is described in Supplementary Methods.

### Nonlinear regression

The % ADM versus time profile data were fit to a sigmoidal maximum effect (E_max_) model using Monolix software (version 2024R1). The E_max_ parameter (i.e., maximum possible effect) was determined from the fit.

### ADM index

Molecular index of ADM (ADMI) was modified from Aney and colleagues (34). Two ADMI indices were developed: ADMI_Up_ and ADMI_Down_. ADMI_Up_ was determined from the mean expression of 19 genes that increased during ADM, and ADMI_Down_ was calculated from the mean expression of 17 genes decreased during ADM (Supplementary Table S2; ref. 34).

### Statistical analysis

Gene set enrichment analysis (GSEA) using the GSEA software v 4.3.2 (Broad Institute) was applied to assess pathway enrichment (35). Gene sets were obtained from the Molecular Signatures Database v 4.3.2. All additional statistical analysis was completed on GraphPad Prism v 10. Intergroup differences in gene expression levels were examined using a two-tailed Mann–Whitney U test. A *P* < 0.05 was considered significant.

## Results

### Cohort description

The demographic details of the 69-donor cohort of normal, pancreatic acinar tissues are listed in Supplementary Table S1. All acinar cell samples were procured from pancreatic islet transplantation centers. Specimens were typically received at the University of Florida research laboratories within 24 hours of the surgery. The acinar cells were obtained from donors who died with a healthy pancreas with the exception of nine diabetics (three type 1 and six type 2). The donors were primarily male (68%), with a median age of 47 years and had representation from three different self-reported races, 17 Black donors (25%), 20 Hispanic donors (29%), and 32 White donors (46%). Fourteen of the samples were reported in our prior study (GSE179248; ref. 14). To our knowledge, this cohort represents the largest and most racially diverse group of normal human pancreases studied for transcriptomics and ADM.

### Subtyping normal, human acinar cells

**Figure 1.**
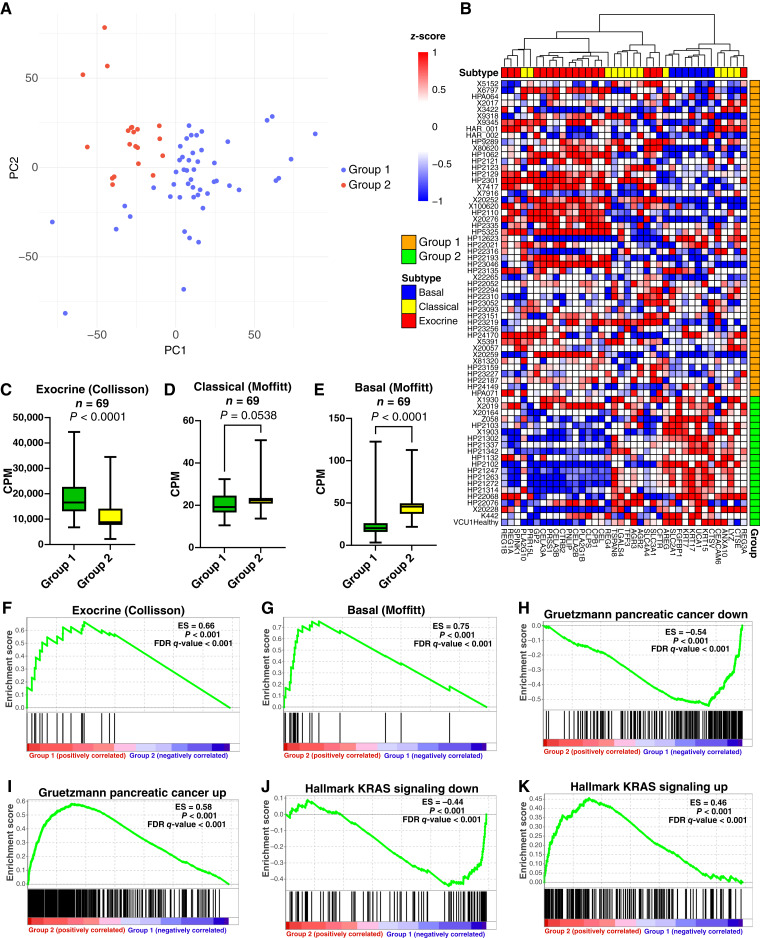
Normal pancreatic acinar cells display molecular subtypes. Sixty-nine specimens of primary, normal pancreas underwent bulk transcriptomic sequencing. **A,** The gene expression data were subjected to spectral clustering analysis, and the resulting data were plotted using PCA. The analysis showed the separation of two different subtypes of donors referred to as group 1 and group 2. **B,** Supervised hierarchical clustering of group 1 and group 2 samples using the basal (Moffitt), classical (Moffitt), and exocrine (Collisson) subtype genes sets. Mean expression of the exocrine (**C**), classical (**D**), and basal (**E**) subtypes as stratified by group. GSEA was performed on the bulk RNA transcriptomic data from the uncultured, normal human pancreatic acinar cells from 69 donors. Shown are the data from the group 1 and group 2 subtypes using the (**F**) exocrine, (**G**) basal, (**H**) Gruetzmann PDAC down and (**I**) Gruetzmann PDAC up, and (**J**) KRAS down and (**K**) KRAS up gene sets. GSEA for (**K**) ADMI_Up_ and ADMI_Down_ gene sets in the group 1 and group 2 classified samples. Mean ± SD. Two-tailed Mann–Whitney U test. CPM, counts per million; ES, enrichment score; PC, principal component.

We initially focused on analyzing the transcriptional profiles of 69 uncultured day 0 acinar samples, all of which passed quality control. The RNA-seq data of the 500 most variably expressed genes from the uncultured, day 0 samples were analyzed using spectral clustering to determine whether any inherent molecular differences exist among the samples prior to culturing. The spectral clustering analysis classified the data into two groups that we arbitrarily describe as group 1 (49 donors) and group 2 (20 donors; Supplementary Table S3). Spectral clustering results were visualized using principal component analysis (PCA), revealing two distinct data clusters (Fig. 1A).

We next asked whether these data classify by the genes common to those used to subtype PDAC (22, 24, 25), including the basal (Moffitt), classical (Moffitt), and exocrine (Collisson) subtypes (Supplementary Table S4). Thirty-eight expressed genes were associated with the dataset. Unsupervised hierarchical clustering for these genes (seven basal, 13 classical, and 18 exocrine-like) revealed that the genes that are primarily acinar (i.e., digestive enzymes) were abundantly expressed in group 1 (decreased in group 2), whereas those genes classifying as cellular adhesion, migration, and structure were highly expressed in group 2 (reduced in group 1, Fig. 1B). Stratifying the mean expression of exocrine, classical, and basal subtypes by group revealed that exocrine genes decreased and basal and classical genes increased in group 2 (Fig. 1C–E). To delve deeper into the nature of the subtypes, GSEA was applied on the transcriptomic data. Like the heatmap (Fig. 1A), the group 1 subtype was positively enriched for the exocrine gene set (Collisson; Fig. 1F), whereas the group 2 subtype positively enriched for basal (Moffitt; Fig. 1G). The GSEA for classical (Moffitt) gene set was not enriched (Supplementary Fig. S2).

GSEA was performed on 7,233 curated gene sets on the group 2 versus group 1 data. The top enriched gene set in the group 1 phenotype was Gruetzmann pancreatic cancer-down, which was negatively enriched in group 1 (Fig. 1H). The Gruetzmann pancreatic cancer-up gene set was among the most positively enriched for the group 2 subtype (Fig. 1I). KRAS signaling down was the most negatively enriched among the 50 hallmark gene sets for the group 1 subtype (Fig. 1J), and KRAS signaling up was among the most positively enriched gene sets for the group 2 subtype (Fig. 1K). Because of their distinguishing gene expression profiles, group 1 will hereafter be referred to as the exocrine-resembling tissue (ERT) subtype and group 2 as the classical/basal (C/B) subtype. We conclude that normal, human pancreatic acinar cells may be grouped into subtypes like those used to classify PDAC.

### ADM, donor demographics, and subtype classification

**Figure 2.**
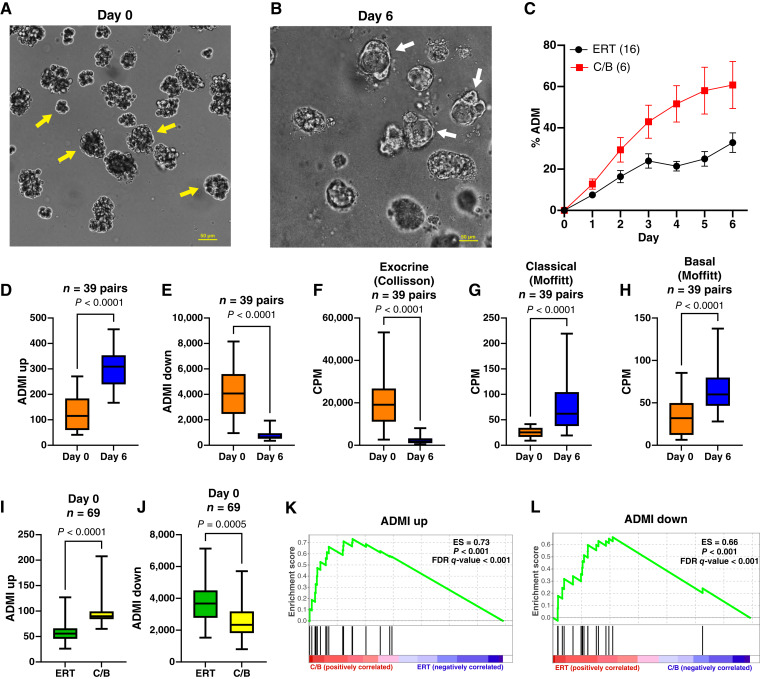
Degree of ADM in normal pancreatic acini differs by culture time. Acinar cells from a 37-year-old Black male were cultured on Matrigel in serum-free conditions and imaged using brightfield microscopy at day 0 (**A**) or following 6 days of culture (**B**). Yellow arrows denote acinar cells, whereas White arrows indicate ductal cells. 20× magnification. **C,** The percentage of ADM was quantified by daily microscopic cell counting in the ERT and C/B subtypes. ADMI was calculated for genes that are (**D**) increased (ADMI_Up_) or (**E**) reduced (ADMI_Down_) following 6 days of culture. Shown are the expressions of (**F**) exocrine (Collisson), (**G**) classical (Moffitt), and (**H**) basal (Moffitt) as stratified by group in uncultured (day 0) and cultured (day 6) cells. ADMI_Up_ and ADMI_Down_ assignment as stratified by the day 0 subtype classification and presented as mean expression levels (**I** and **J**) and GSEA (**K** and **L**). Mean ± SD. Two-tailed Mann–Whitney U test. CPM, counts per million; ES, enrichment score.

*In vitro* ADM transdifferentiation experiments were performed on 39 pairs of acinar (uncultured, day 0) and ADM transdifferentiated (6 days of culture) donor specimens. As in our previous work with human acinar tissues (14), the 39 pairs of acinar cells cultured in serum-free conditions underwent transdifferentiation from an acinar to a ductal state (Fig. 2A and B). The % ADM for the C/B subtype was enhanced compared with ERT (Fig. 2C). Bulk transcriptomic sequencing was performed on the RNA extracted from all 39 pairs of day 0 and day 6 cultures. As an indication of the degree of ADM that each sample underwent, we applied a variation of the ADMI (34). The mean expression of a subset of genes that increased with ADM (ADMI_Up_) and another subset of genes with decreased expression following ADM (ADMI_Down_) were assigned (Supplementary Table S2). ADMI_Down_ genes are primarily exocrine genes (pancreatic enzymes and genes regulating translation), and ADMI_Up_ genes encode for proteins that are involved in a variety of functions, including epithelial cell differentiation and function and other developmental processes.

As anticipated, ADMI_Up_ genes increased and ADMI_Down_ genes decreased following 6 days of ADM transdifferentiation (Fig. 2D and E). Comparing the expression of the exocrine, classical, and basal genes, in day 0 uncultured samples and those following 6 days of ADM revealed that the exocrine gene expression was reduced during ADM and the classical and basal expression increased with ADM (Fig. 2F–H). When stratified by subtype in the 69 uncultured day 0 samples, the expression of the exocrine ADMI_Up_ genes was higher in C/B compared with ERT, whereas the opposite was observed for the ADMI_Down_ genes (Fig. 2I and J). In the GSEA, the ADMI_Up_ gene set was positively enriched in C/B (Fig. 2K), and the ADMI_Down_ gene set was positively enriched in ERT (Fig. 2L). The ADMI_Up_ and ADMI_Down_ indices were validated on a publicly available dataset of caerulein-induced pancreatitis in mice (GSE65146). As anticipated, the values for ADMI_Up_ increased with the times after caerulein injection and ADMI_Down_ values decreased (Supplementary Fig. S3). We conclude that during experimental ADM of normal pancreas, there is a shift from the ERT gene signature to the C/B subtype. Morphologic ADM is increased in the C/B subtype, and the ADMI may be used to gauge the propensity for ADM in uncultured acinar cells.

### Impact of ancestry on ADM

**Figure 3.**
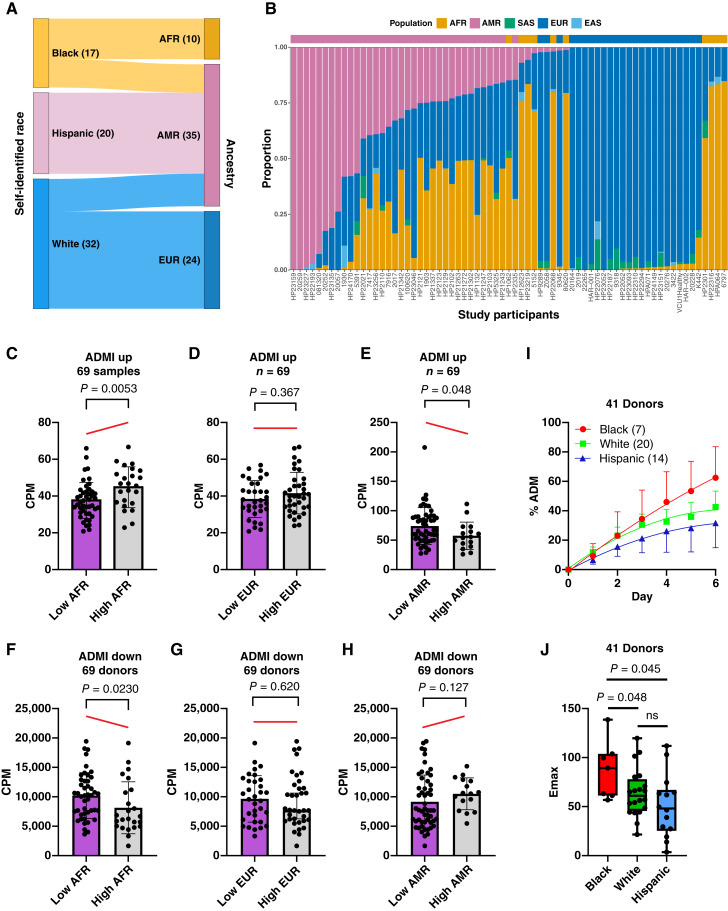
Genetic ancestry and race correlate with degree of ADM and molecular subtypes. **A,** The relationship between self-identified race/ethnicity and inferred continental genetic ancestry for the cohort of normal pancreatic acini from 69 donors. **B,** Inferred continental admixtures in the cohort with the PCA-based continental ancestry in the top track. Correlation plots were made for the proportion of AFR (**C** and **F**), EUR, (**D** and **G**), or AMR (**E** and **H**) genetic ancestry. Data are shown for ADMI_Up_ (**C–E**) and ADMI_Down_ (**F–H**). The red line illustrates the relative shift in mean CPM values between groups, providing a visual cue for the directionality of the observed differences. Pancreatic acinar cells from 41 deceased organ donors of differing self-identified races were cultured and monitored for the degree of ADM by microscopic duct counts over a 6-day period. **I,** The acinar cells from 41 donors were cultured in serum containing media, and the percentage of ducts and acinar clusters was microscopically counted as described in Supplementary Methods. The mean ± SEM of the % ADM from the 41 donor’s acinar specimens are plotted. The kinetic data for the mean of the 41 donors of each self-identified race were modeled to a sigmoid E_max_ model. The mean ± SD is plotted for (**J**) E_max_. Two-tailed Mann–Whitney U test. CPM, counts per million; EAS, East Asian, SAS, South Asian.

Our next objective was to determine whether a relationship exists between the donors’ genetic ancestry and ADM. Ancestry analysis was performed on the RNA transcriptomic data of the 69 donor’s acinar samples (33). The Sankey diagram shows the comparison between the self-identified race and genetic ancestry (Fig. 3A). The admixture graph shows the correlation between ancestry (Fig. 3B) and the super population that was determined by PCA plots (Supplementary Table S1; Supplementary Fig. S4). To relate genetic ancestry to ADM, the ADMI data were stratified by the ancestry admixture. A 33% cutoff was applied to distinguish low and high ancestral admixtures. For AFR ancestry, the ADMI_Up_ expression increased with increasing AFR admixture (Fig. 3C). ADMI_Up_ values were unchanged for EUR ancestry (Fig. 3D) and decreased with increasing AMR admixture (Fig. 3E). For the ADMI_Down_ comparisons, the values were decreased, unchanged, and increased with increasing admixture in the AFR, EUR, and AMR donors, respectively (Fig. 3F–H).

The degree of morphologic ADM was assessed by microscopic duct counting over the 6-day period of *ex vivo* ADM. The kinetic data were fit to a nonlinear regression model to account for the determination of parameters such as E_max_. The degree of morphologic ADM and E_max_ decreased in the order of Black > White > Hispanic donors (Fig. 3I and J). The kinetic curves for the individual donors are presented in Supplementary Fig. S5. We conclude that those normal pancreatic acinar cell specimens with a higher AFR ancestral admixture undergo more morphologic ADM and have a higher molecular propensity for ADM compared with specimens obtained from donors of EUR or AMR descent.

### Validation in an independent cohort of normal human pancreata

**Figure 4.**
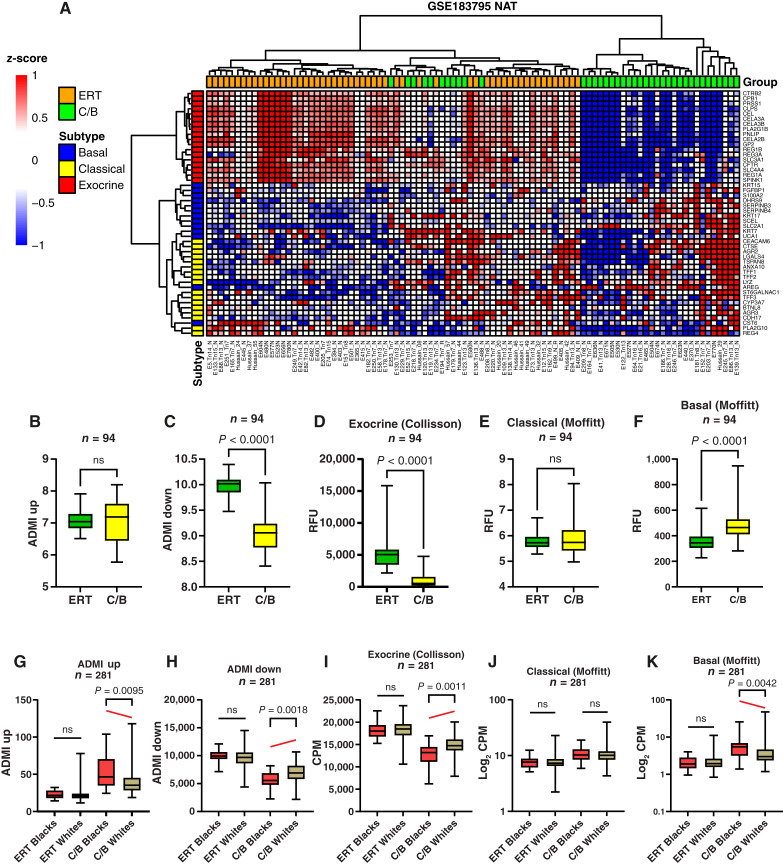
Validation of ADM indices in independent cohorts of normal pancreata. **A,** Supervised hierarchical clustering of ERT and C/B samples using the exocrine (Collisson), classical (Moffitt), and basal (Moffitt) subtype gene sets in the GSE183795 validation set. **B,** ADMI_Up_, (**C**) ADMI_Down_, (**D**) exocrine (Collisson), (**E**) classical (Moffitt), and (**F**) basal (Moffitt) as stratified by ERT and C/B subtype for NAT in the GSE183795 validation set. **G,** ADMI_Up_, (**H**) ADMI_Down_, (**I**) exocrine (Collisson), (**J**) classical (Moffitt), and (**K**) basal (Moffitt) stratified by ERT and C/B subtype and self-identified race in the GTEx validation cohort of 281 normal pancreata. Mean ± SD. Two-tailed Mann–Whitney U test. CPM, counts per million; RFU, relative fluorescent unit.

The subtype-defining gene expression profiles in normal pancreas were validated in two independent cohorts: one with 94 normal adjacent to tumor (NAT) from GSE183795 (36) and another 281 normal pancreas specimens from the GTEx portal (https://www.ncbi.nlm.nih.gov/gap/). Hierarchical clustering of the basal, classical, and exocrine gene expression revealed the presence of two clusters of data for the NAT samples (Fig. 4A). The heatmap showed that the ERT subtype exhibited high expression of exocrine genes, whereas the C/B subtype displayed increased expression of classical and basal markers. ADMI_Up_ and classical gene expression were unchanged between the two subtypes (Fig. 4B and E). ADMI_Down_ and exocrine gene expression decreased in the C/B subtype (Fig. 4C and D), whereas basal expression increased in C/B (Fig. 4F). Grade and stage of the primary PDAC did not influence NAT subtype assignment (Supplementary Fig. S6).

The GTEx cohort includes 281 tissues obtained from donors who remained on a ventilator upon death and contained no autolysis or pancreatitis. The heatmap from the GTEx data also revealed two subtypes: ERT with high exocrine gene expression and C/B with increased classical and basal gene expression (Supplementary Fig. S7). Comparisons between self-identified race and molecular subtype were performed within each subtype. Only Black individuals in the C/B subtype showed statistically significant differences, with ADMI_Up_ being increased and ADMI_Down_ being reduced (Fig. 4G and H). The expression of exocrine, classical, and basal genes was increased, unchanged, and reduced, respectively, in Black C/B compared with White C/B donors, whereas no differences were apparent in the expression of these genes between the races for the ERT classification (Fig. 4I–K).

**Figure 5.**
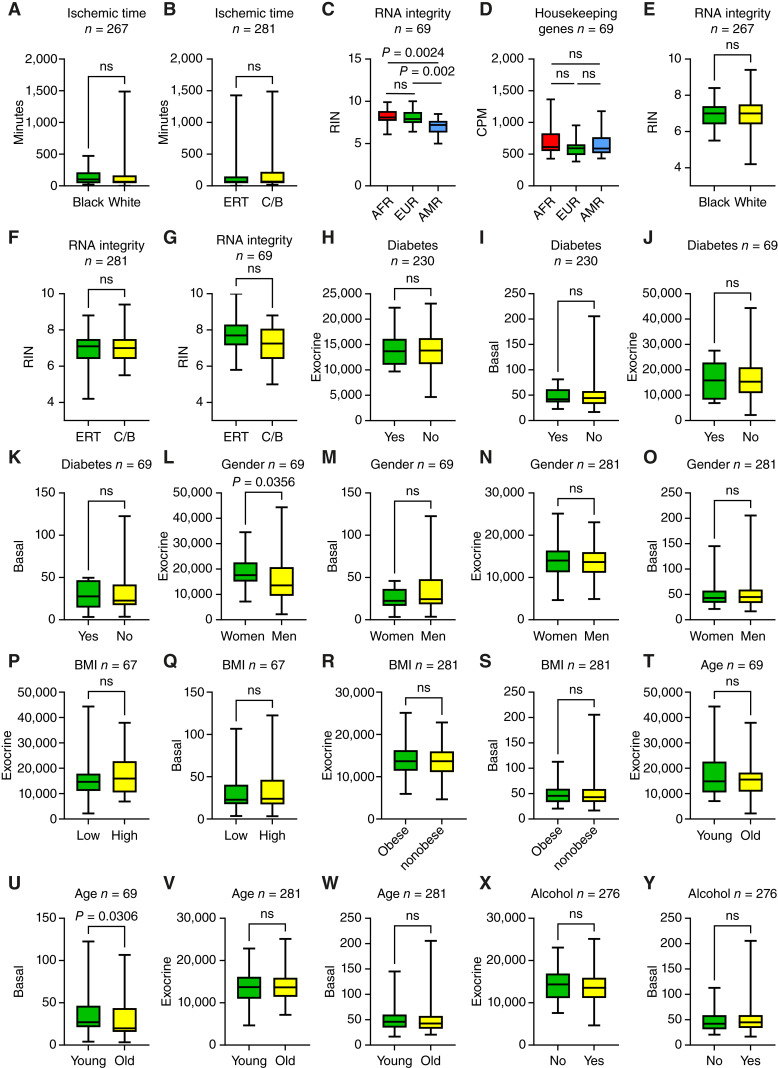
Association of clinical and processing variables with subtype and ancestry classification. Metadata were mined for the cohort of 69 uncultured normal pancreatic acinar cells (**C**, **D**, **G**, **J–M**, **P**, **Q**, **T**, and **U**) and the normal pancreas from the GTEx validation cohort (**A**, **B**, **E**, **F**, **H**, **I**, **N**, **O**, **R**, **S**, and **V–Y**). Mean ± SD. Two-tailed Mann–Whitney U test. ns, not significant. BMI, body mass index; CPM, counts per million.

To rule out the possibility of technical or biological artifacts contributing to our findings, we compared the gene expression data with available metadata on the specimens. In the GTEx validation cohort, neither race nor subtype was influenced by warm ischemic time (Fig. 5A and B). In the 69-sample cohort, RIN was reduced in the AMR ancestry compared with AFR and EUR ancestries (Fig. 5C); however, the expression of 20 reference genes (Supplementary Table S5) showed no statistically significant differences with ancestry (Fig. 5D). RNA integrity did not affect race or subtype (Fig. 5E and F). For the cohort of 69 purified acinar cell samples, no statistical differences were observed between RNA integrity and subtype (Fig. 5G). Comparisons of potential confounders (age, gender, body mass index, diabetes, or alcohol use) revealed no statistically significant differences (Fig. 5H–Y) with the exception of gender (Fig. 5L) and age (Fig. 5U) in the 69-sample cohort. For these comparisons, the observed fold changes were modest (<1.5 fold), and these trends were not recapitulated in the larger GTEx cohort (Fig. 5N and V). We conclude that potential confounders such as diabetes, obesity, age, gender, or specimen processing variables did not influence the distribution of samples across subtype classification or ancestry/race.

### Pathways downstream of *KRAS* are activated in a subset of normal human pancreas

**Figure 6.**
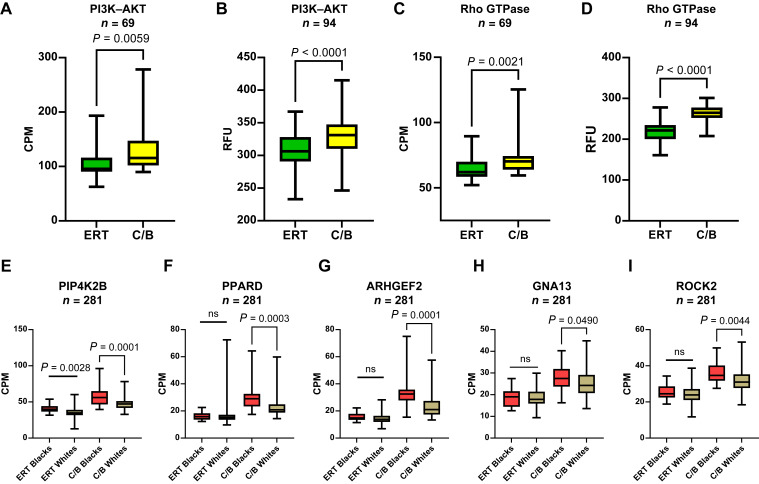
Activation of PI3K–AKT and Rho GTPase pathways in normal pancreas. The data from uncultured day 0 normal pancreatic acinar cells (**A** and **C**) or NAT of patients with PDAC from GSE183795 (**B** and **D**) were stratified by subtype for the PI3K–AKT (**A** and **B**) or Rho GTPase (**C** and **D**) gene sets. Individual expression values for genes regulated by the (**E** and **F**) PI3K–AKT (PIP4K2 and PPARD) or (**G–I**) Rho GTPase (ARHGEF2, GNA13, and ROCK2) pathways stratified by subtype and self-identified race in the GTEx database. Mean ± SD. Two-tailed Mann–Whitney U test. CPM, counts per million; RFU, relative fluorescent unit.

Lo and colleagues (37) used a KLF4 mouse model of ADM to discover DNA methylation patterns of exocrine genes such as PI3K and Rho/Rac/Cdc42 GTPase (R/R/C GTPase) that drive ADM in the absence of oncogenic driver mutations. As our findings show basal signature expression in normal pancreas with wild-type KRAS, we investigated whether the activation of PI3K and R/R/C GTPase pathways is observed in the C/B and ERT subtypes. Comparing the mean expression of the PI3K–AKT and R/R/C GTPase gene sets (Supplementary Table S6) between ERT and C/B revealed an increase in the mean expression of both gene sets in the C/B subtype in the normal acinar (Fig. 6A and C), GSE183795 (Fig. 6B and D), and GTEx (Supplementary Fig. S8A and S8B) cohorts. We compared the expression of six genes with increased expression in ADM in the absence of Kras mutations (37), including PI3K-related AKT1, PIP4K2, and PPARD, as well as R/R/C GTPase–related ARHGEF2, GNA13, and ROCK2 in the GTEx cohort stratified by self-identified race. The expression of five of the six genes was increased in C/B, Black donors (Fig. 6E–I). PI3K–AKT and R/R/C GTPase increased in C/B compared with ERT (Supplementary Fig. S8C and S8D). Pathways downstream of KRAS are activated in normal human pancreatic acinar cells that classify as the C/B subtype and are elevated in Black compared with White donors that subtype as C/B.

### Inflammatory and immune signaling programs are enriched in the C/B subtype of normal pancreas

**Figure 7.**
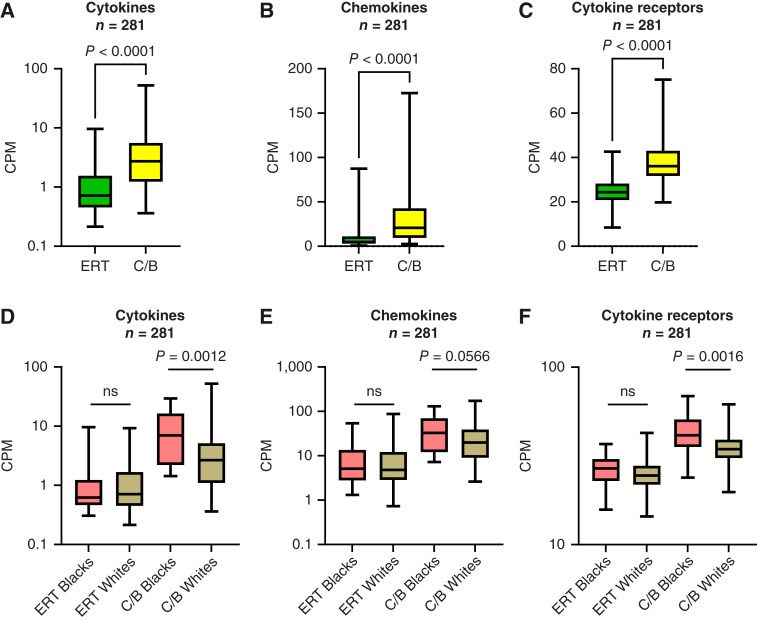
Immune signaling is activated in the C/B subtype of normal pancreas and further enhanced in Black donors. The mean expression of (**A**) cytokines, (**B**), chemokines, and (**C**) cytokine receptors in normal pancreas tissue from the GTEx cohort. Mean expression values for the (**D**) cytokines, (**E**) chemokines, and (**F**) cytokine receptors stratified by subtype and self-identified race. Mean ± SD. Two-tailed Mann–Whitney U test. CPM, counts per million.

A recent single-cell analysis of mouse pancreas using an injury model revealed that acute pancreatitis triggers an immune response in acinar cells that is accompanied by the loss of normal acinar identity (34). To assess the contribution of immune components to subtype classification in the normal pancreas, we analyzed cytokine, chemokine, and cytokine receptor expression in the GTEx cohort, which represents whole pancreas tissue and retains immune cell populations that are presumably absent from purified acinar samples. The mean expression of cytokines (CXCL8, IL1B, IL6, LIF, and TNF), chemokines (CCL2, CCL20, CXCL1, CXCL2, and CXCL5), and cytokine receptors (CSF2RA/B, IL1R1, LIFR, and TNFRSF1A/B) increased in C/B compared with ERT (Fig. 7A–C; Supplementary Fig. S9). Moreover, the expression of the immune components was unchanged in ERT Black versus White donors but increased in C/B Black compared with White donors (Fig. 7D–F). These findings suggest an immune component to the subtype classification in normal pancreas that is independent of KRAS mutation.

## Discussion

It is well established that PDAC classifies into molecular subtypes (22–26) and that PDAC subtype classification has implications in the prognosis and treatment outcomes for patients. The most significant finding of our study is the apparent presence of PDAC subtypes in otherwise normal human acinar cells and pancreatic tissues. We describe two subtype classifications: ERT, characterized by high acinar gene expression with low classical and basal signatures, and C/B, which displays reduced exocrine and increased classical and basal gene expression (Figs. 1B and 4A). Although it is unremarkable that normal acinar cells would retain an exocrine signature, 29% of the normal acinar tissues identified with the C/B subtype. Moreover, approximately 45% of the NAT and normal pancreas from two independent validation cohorts classify as C/B. Although an explanation for this phenomenon is not clear, it does not seem to be due to the presence of mutations common to PDAC development as two of the three cohorts in our study consist of normal pancreases from healthy donors, and all 69 samples in the purified acinar cell cohort contained wild-type *KRAS* at codon 12 (Supplementary Table S1). Epigenetic factors regulate ADM in mice independently of mutant *KRAS* (37) and may influence subtype classification in human acinar cells.

Given the limitations of mouse models in recapitulating human ADM biology, we conducted our study on primary human acinar tissues. Aside from prior work (10, 12, 38–40), this represents an approach that is seldom used in this field. As demonstrated here, human tissue models provide direct insights into human pancreatic biology and retain the donor heterogeneity present in the general population. However, the use of human tissue also presents challenges that if not properly addressed may introduce bias. To improve rigor, we confirmed our results in two independent cohorts with sufficient power: GSE183795 (*n* = 94) and GTEx (*n* = 281). Pancreatic acinar cells are highly sensitive to processing-related variations. Purified acinar cells undergo spontaneous ADM due to warm ischemia time, hypoxia, or delay of obtention of the tissue. Metadata on the tissues were mined to address potential contributing factors for the observed differences in gene expression among subtype and race. Warm ischemia time and RNA integrity did not affect subtype classification of the tissues or influence the expression difference with respect to ancestry/race (Fig. 5). Another possible contributor to the gene expression differences in the dataset is the influence of clinical confounders such as age, obesity, diabetes, gender, and alcohol use. However, none of these factors influenced subtype assignment or the observed differences across ancestry/race (Fig. 5). Interestingly, among all of the clinical and processing-related variables studied, only subtype and ancestry/race showed consistent statistically significant differences within the cohorts.

Another interesting and unexpected finding was the profound reduction in acinar gene expression in the C/B normal acinar (Fig. 1B), NAT (Fig. 4A), and normal pancreas (Supplementary Fig. S7) specimens. This reduction in acinar genes correlated with the reduced expression of key transcriptional drivers of acinar gene expression, including *MIST1* (*BHLHA15*), *PTF1A*, and *RBPJL* (Supplementary Fig. S10). Maintaining acinar cell identity attenuates *Kras*-induced tumorigenesis in mice and reexpressing *Ptf1a* in precancerous lesions induces quiescence and redifferentiation in mice (41). Therefore, pancreatic tumors with a more exocrine-like phenotype are associated with better outcomes, including patients whose PDAC showed high expression of an acinar cell–derived signature (27). Experimental therapies that promote acinar identity through inhibition or reversal of ADM (15, 16) are potential therapeutic approaches for treating PDAC that warrant further development.

Variant-calling tools were used to assign the proportion of genetic ancestry to each donor’s acinar specimen. This allowed us to directly compare donors’ ancestry to other quantitative indices, such as ADMI. Analysis of the Surveillance, Epidemiology, and End Results data showed that the trend for both incidence and mortality of PDAC decreased in the order of non-Hispanic Blacks > non-Hispanic Whites > Hispanics (42). Interestingly, morphologic ADM (Fig. 3I and J) and correlations between the percentage ancestry and ADM in specimens of completely normal pancreatic acinar cells parallel the incidence and mortality of patients with PDAC with respect to ancestry/race (Fig. 3C–H). When the gene expression data were stratified by both self-identified race and subtype, specimens from Black individuals who aligned with the C/B subtype had the most aggressive molecular phenotype, i.e., reduced exocrine, increased basal/classical expression, lower expression of genes reduced during ADM, increased expression of genes increased during ADM (Fig. 4G–K), increased activation of PI3K–AKT and Rho GTPase pathways (Fig. 6E–I), and increased activation of immune signaling (Fig. 7D–F). These associations suggest a potential biological basis for the disparities observed in PDAC incidence among individuals of AFR descent that warrants further investigation.

We report on the largest study of its kind to examine the gene expression profiles and molecular indices of ADM using primary normal, human pancreatic tissues. Robust quantitative data were generated that to our knowledge have not been reported in normal human acinar specimens, including using ancestry admixture as a variable. Our findings suggest that a permissive state exists in normal pancreas that predisposes acinar cells to undergo early reprogramming and ADM. Moreover, it is tempting to speculate that individuals whose normal pancreas subtypes as C/B may be predisposed to developing PDAC following acquisition of mutations in *KRAS* and tumor suppressors.

## Supplementary Material

Supplementary Data Supplemental Data and Methods

Supplementary Figure S1 Figure S1. Principal component analysis (PCA) of bulk RNA-seq data from normal pancreatic acinar specimens.

Supplementary Figure S2 Figure S2. GSEA of Group 1 and Group 2 data for 69 normal acinar specimens.

Supplementary Figure S3 Figure S3. Validation of ADMI on independent, data set.

Supplementary Figure S4 Figure S4. PCA based continental-level ancestry inference done with RAIDS software.

Supplementary Figure S5 Figure S5. ADM transdifferentiation kinetics as modeled to sigmoid Emax model.

Supplementary Figure S6 Figure S6. Association between tumor stage, grade and subtype classification.

Supplementary Figure S7 Figure S7. Heatmap of gene expression from 281 normal pancreas.

Supplementary Figure S8 Figure S8. Activation of PI3K-AKT and Rho GTPase pathways in Group 2 normal pancreas.

Supplementary Figure S9 Figure S9. Immune components are increased in C/B subtype of GTEx cohort.

Supplementary Figure S10 Figure S10. Increased expression of acinar transcription factors in Group 1 of NAT from independent cohort.

Supplementary Table 1 Supplemental Table 1. Donor Demographics

Supplementary Table 2 Supplemental Table 2. Gene signature for ADMI Up and ADMI Down

Supplementary Table 3 Supplemental Table 3. Results of spectral clustering

Supplementary Table 4 Supplemental Table 4. Results of Ancestry, Subtype and ADM Indices

Supplementary Table 5 Supplemental Table 5. Twenty housekeeping genes for Figure 5D

Supplementary Table 6 Supplemental Table 6. PI3K-AKT and Rho GTPase gene set used in Figure 5

## Data Availability

All curated datasets were posted to the GEO repository under accession number GSE295071. Other data generated in this study are available upon request from the corresponding author.
