# Supplementary material for: Transcriptomic Analyses of Normal Human Pancreata Reveal the Presence of Cancer Subtypes that Correlate with Acinar Ductal Metaplasia and Donor Ancestry
Source: Cancer Res Commun. 2026 Jan 21;6(1):165–77. doi: 10.1158/2767-9764.CRC-25-0411 (PMC12820465; doi:10.1158/2767-9764.CRC-25-0411)
Supplement: Supplementary Data — Supplemental Data and Methods [file crc-25-0411_supplementary_data_suppsd.docx]

**Supplemental methods and data**

**Transcriptomic analyses of normal human pancreata reveal the presence of cancer subtypes that correlate with acinar ductal metaplasia and donor ancestry**

**Corey M. Perkins^1,10*^, Jinmai Jiang^1,10*^, Kalyanee Shirlekar^2,10^, Zachary Greenberg^1^, Md Abu Talha Siddique^1,10^, Jason Brant^2,10^, Kiley Graim^3^, Mei He^1^, Sarah Kim^1^, Diana J. Wilkie^4,10^, Bo Han^5,10^, Jamel Ali^6,10^, Pascal Belleau^7,8^, Astrid Deschênes^8^, Alexander Krasnitz^7,8^, Mazhar Kanak^9^ and Thomas D. Schmittgen^1,10 †^**

**^1^Department of Pharmaceutics, College of Pharmacy, ^2^Department of Biostatistics, ^3^Department of Computer & Information Science & Engineering, Herbert Wertheim College of Engineering, University of Florida, Gainesville FL, ^4^Department of Behavioral Nursing Science, College of Nursing, University of Florida, Gainesville, FL. ^5^Department of Surgery, University of Southern California, Los Angeles, CA, USA. ^6^Department of Chemical and Biomedical Engineering, FAMU-FSU College of Engineering, Tallahassee, FL,^7^Simons Center for Quantitative Biology, ^8^Cancer Center, Cold Spring Harbor Laboratory, Cold Spring Harbor, New York. ^9^Department of Transplant Surgery, Virginia Commonwealth University, Richmond, VA. ^10^Florida-California Cancer Research Education and Engagement Health Equity Center.**

**^*^These authors contributed equally to this work.**

**^†^Corresponding author: Thomas D. Schmittgen,** [**tschmittgen@ufl.edu**](mailto:tschmittgen@ufl.edu)**. Cancer Genetics Research Complex, 2033 Mowry Road, Gainesville, FL 32610. 352-273-8028.**

**Keywords: Pancreatic cancer, acinar ductal metaplasia, ADM, cancer subtypes, health disparities, organoids, ancestry.**

**Conflict of interest statement: None of the authors report a conflict of interest.**

**Abbreviations: ADM, acinar ductal metaplasia; ADMI, acinar ductal metaplasia index; ERT, exocrine resembling tissue; C/B classical/basal subtype; GSEA, gene set enrichment analysis; PDAC, pancreatic ductal adenocarcinoma; AFR, African; EUR, European; AMR, Ameridigenous; EAS, East Asian; SAS, South Asian; NAT, normal adjacent to tumor; SEER, Surveillance, Epidemiology, and End Results;** **R/R/C, Rho/Rac/Cdc42.**

**Running title: PDAC molecular subtypes in normal human pancreata**

**EXTENDED METHODS**

Human pancreatic acini. Pancreatic islet cells comprise only 2-3% of the total pancreatic cell mass. Islet cells were purified from the total pancreatic cell digest by a density gradient purification process using a COBE 2991 cell processor. Human acinar cells (1.100 – 1.115g/ml) have a higher density than human islets (1.075-1.100g/ml). After a density gradient centrifugation, islets are collected in earlier fractions, and acinar cells are collected in the lower fractions. Finally, the cells collected in the last fraction from the COBE bag consist of greater than 99% acinar cells, which are used for the culture of acinar cells and experiments. Human islets obtained in the earlier fractions are combined for transplantation or other downstream experiments. Primary human acinar cells from deceased organ donors were shipped on blue ice in PIM-T media (1% PIM-G; 2.5% AB serum; 10 µg/mL ciprofloxacin hydrochloride; 100 µg/mL trypsin inhibitor from Glycine Max). Acini were received from Prodo Laboratories pancreatic islet transplantation centers (Aliso Viejo, CA), Network for Pancreatic Organ Donors with Diabetes (nPOD), University of Miami and Virginia Commonwealth University. The study protocol was reviewed and approved by the University of Florida Institutional Review Board (IRB201902530). Donor demographics of the 69 organ donors are provided in **Supplemental Table 1**. Self-reported race of the donors is listed as Black, White and Hispanic whereas ancestral admixture is defined as African (AFR), European (EUR) or Ameridigenous (AMR).

Acinar cell culture. Acinar cells were cultured as previously described (1). Briefly, upon receipt, acinar cells were placed on ice prior to transfer to the biological safety cabinet for processing. The PIM-T media/cell suspension was sequentially pipetted through sterile cell strainers (500, 300, 200 and 100 µm). Cells were passed through the strainer’s grid by gently scratching the membrane. Cells were then centrifuged at 720×g for 2 minutes at 4° C. The supernatant was discarded, and the acinar cell pellet was resuspended in a 5 ml in plating media (1:1 mixture of DMEM 4.5 g/L D-Glucose and F12K Nutrient Mixture) to the desired seeding density (approximately 800 acinar clusters per well of a 48-well plate). The cell suspension was mixed in a 1:1 ratio with ice-cold, growth-factor reduced Matrigel (Corning) and 200 µl was plated per well of a tissue culture treated 48-well plate. The plates were incubated at 37° C for 30 minutes to solidify the Matrigel and 300 µl of pre-warmed feeding media (DMEM 4.5 g/L D-Glucose, F12K Nutrient Mixture, and 0.1 mg/ml soybean trypsin inhibitor). Cells that were counted for the percent of ADM were cultured in (Waymouth’s (1X) Medium, 20 μg/ml dexamethasone, 10% fetal bovine serum, 0.1 mg/ml soybean trypsin inhibitor, 10,000 U/ml penicillin, and 10,000 μg/ml streptomycin) in 96 well plates. Adhesive grids were placed on the bottom of culture plates prior to cell plating for daily microscopic counting of acinar and ductal cells to obtain a percent ADM (1). Cells were plated as described above in Matrigel for both Day 0 (arrival day) and Day 6 collection, however the Matrigel was immediately removed from the cells after plating as described (2).

RNA isolation. Matrigel was removed from the cells as previously described (2). The resulting cell pellets were lysed in 700 μL of Trizol reagent. Total RNA was isolated from the Trizol lysate using the Qiagen miRNeasy Mini Kit (Qiagen, Cat # 217004) according to the manufacturer's protocol. RNA was eluted in 30 μL of molecular-grade water containing 1 ul of RNase Inhibitor (NEB), and its concentration was quantified using a NanoDrop spectrophotometer. RNA integrity was assessed using the Agilent TapeStation Bioanalyzer. Only RNA samples with an RNA Integrity Number (RIN) greater than 5.0 were used for cDNA and next-generation sequencing (NGS) library preparation (**Supplemental Table 1**).

Library Preparation for Illumina NovaSeq Sequencing. RNA sequencing (RNA-seq) libraries were prepared from 60 ng of total RNA, with each sample processed individually, following the manufacturer's protocol for the NEBNext Ultra II Directional RNA Library Prep Kit for Illumina (NEB #E7760S, New England Biolabs, USA). mRNA was first enriched from total RNA using NEBNext Poly(A) mRNA Magnetic Isolation Module (NEB #E7490) and subsequently fragmented by incubation at 94°C. First-strand cDNA synthesis was carried out using reverse transcriptase and random primers. This was followed by second-strand cDNA synthesis, end-repair, and dA-tailing. Illumina-specific adaptors were then ligated to the cDNA fragments. The adaptor-ligated DNA was enriched and indexed using NEBNext Multiplex Oligos for Illumina (96 Unique Dual Index Primer Pairs, New England Biolab), with 13 cycles of amplification. Library purification was performed using NEBNext Sample Purification Beads (New England Biolabs).

The library size and mass were assessed using the Agilent DNA1000 TapeStation, where a narrow size distribution with a peak around 300 bp was typically observed. Library quantification was performed using quantitative PCR (qPCR) with the KAPA Library Quantification Kit (Kapa Biosystems), and amplification was monitored on the ABI7900HT real-time PCR system. For sequencing, libraries were pooled equimolarly and cleaned with 0.8X AMPure beads (Omega Bio-Tek) to reduce adapter-dimer contamination. The pooled library was then diluted to 0.65 nM and sequenced on the Illumina NovaSeq series platform to a target depth of ~50 million reads per sample. A 10B flow cell (2 × 150 cycles) and 10B sequencing reagents were used. The final library loading concentration was 130 pM, with a 5% PhiX spike-in as a control. FastQ files were generated using the BCL2fastQ function in the Illumina BaseSpace portal.

RNA sequence reads. Paired-end sequencing was performed using Illumina NovaSeq series at 2x150 cycle sequencing for 69 samples harvested on Day 0 in 4 batches. RNA-Seq data processing was performed using the nf-core RNA-Seq pipeline v3.12.0 (<https://github.com/nf-core/rnaseq>), a community-driven, reproducible analysis workflow built on the NextFlow DSL2 framework and singularity(3)). The pipeline was executed on the University of Florida’s computing cluster (HiPerGator). Briefly, raw sequencing reads in FASTQ format were first evaluated for quality using FastQC v0.11.9. Adapter sequences were auto detected, and low-quality bases were trimmed using Trim Galore v0.6.7. Reads shorter than 20 bp post-trimming were discarded. Read alignment was performed with STAR v2.7.10a to the reference genome GRCh37 and gene expression quantification was done using RSEM v1.3.1. Checks for mapping quality, duplicate rates, and GC content were conducted. Raw counts were corrected using ComBat-Seq from the sva package v3.52.0 (4) in R programming language to remove batch-induced technical variation.

Filtering of raw counts was performed using edgeR's v4.2.2, (5) default criteria, requiring a minimum of 10 counts in at least 70% of samples. The batch-corrected counts were normalized using the Trimmed Mean of M-values (TMM) method implemented in edgeR which adjusts for differences in library sizes and compositional bias. Further, dimensionality reduction techniques, such as multidimensional scaling (MDS) and principal component analysis (PCA), were applied to the counts. Subsequently, spectral clustering analysis using the package, Spectrum v1.1 (6) was performed on the batch-corrected filtered counts to identify underlying patterns in the data. This analysis revealed two distinct clusters within the log counts per million. All curated data sets were posted to the Gene Expression Omnibus (GEO) repository under accession number GSE295071.

Continental genetic ancestry. Continental genetic ancestry was inferred from the RNA sequencing using the published protocol (7) For the subsequent genotyping and genetic ancestry inference, duplicated mapped reads were removed using Picard version 2.26.11 (<https://broadinstitute.github.io/picard/>). GATK toolkit version 4.2.5.0 (8) was used for reads spanning splice junctions, with mapping quality reassignment (SplitNCigarReads program) followed by base quality score recalibration (BaseRecalibrator program). Allele-specific read counts at genome positions with high single-nucleotide variant frequencies in the 1000 Genomes population reference were extracted using the snp-pileup program in FACETS software package version 0.6.1 (9). The resulting genotypes were pruned to reduce linkage disequilibrium. Continental-level global genetic ancestry was inferred employing Bioconductor RAIDS package in the Bioconductor repository (7). As a result, each donor was assigned one of African (AFR), Ameridigenous (AMR), East Asian (EAS), European (EUR), or South Asian (SAS) continental ancestries. The continental ancestral admixtures of the donors we inferred using an in-house version of RAIDS with the ADMIXTURE software version 1.3.0 in supervised mode (10) as a component. The Sankey diagram was drawn using CRAN networkD3 package version 0.4. CRAN ggplot2 package version 3.5.1 and CRAN cowplot package version 1.1.3 were employed for the barplot of ancestral admixtures. The 3-dimensionsional scatter plots (**Supplementary Fig. 4**) were prepared with CRAN plotly package version 4.10.2.

Kinetic modeling of ADM. The % ADM versus time of culture profiles were modeled to a pharmacodynamic sigmoidal Emax model using Monolix software (version 2024R1). Data from 41 donors were included in the analysis. The model combined the three parameters Emax, EC50, and gamma.

$$A=E_{0}+\frac{EmaxC^{\gamma}}{C^{\gamma}+EC_{50}^{\gamma}}$$

Emax describes the maximum possible effect, EC50 indicates the time point at which ADM reaches 50% of Emax, and gamma determines the steepness of the curves. C was used as a surrogate for time. Monolix output was exported and further analyzed in R (version 4.3). Individual ADM trajectories over time were plotted using the ggplot2 package. Mean ADM curves were generated by self-reported donor group (White, Hispanic and Black) using GraphPad Prism (version 10.4.1).

**Supplemental Figure Legends**

**Supplemental Figure 1. Principal component analysis (PCA) of bulk RNA-seq data from normal pancreatic acinar specimens.** Transcriptomic profiling was performed on 69 primary, normal pancreatic acinar samples. PCA was conducted using 14,346 filtered genes after batch correction, implemented with the PCAtools and ggplot2 packages in R. Samples are stratified by (A) Sequencing Run, and (B) Group. User to assess potential sources of technical variation.

**Supplemental Figure 2. GSEA of Group 1 and Group 2 data for 69 normal acinar specimens.** GSEA was performed on the bulk RNA transcriptomic data from uncultured, normal human pancreatic acinar cells from 69 donors. Shown are the data from the Group 1 and Group 2 subtypes (ratio of Group 2/Group 1) using the classical (Moffitt) gene set.

**Supplemental Figure 3. Validation of ADMI on independent, data set.** Data set GSE183795 was mined for pancreatitis mouse model injected with caerulein. Mice were sacrificed at various time post injections (x-axis) and the degree of pancreatitis. Shown are the ADMI_Up_ (A) and ADMI_Down_ (B) gene expression indices as a function of the degree of pancreatitis.

**Supplemental Figure 4. PCA based continental-level ancestry inference done with RAIDS software.** Examples of two Ameridigenous ancestry assigned patients from the current cohort (patients 1000620 and HP1132) using a PCA-based method. The star marks represent the 1000 Genomes cohort distribution according to their continental ancestry (AFR: green, AMR: orange, EAS: blue, EUR: pink, SAS: yellow). The round mark represents a patient of the current cohort.

**Supplemental Figure 5. ADM transdifferentiation kinetics as modeled to sigmoid Emax model.** Pancreatic acinar cells from 41 deceased organ donors of differing self-identified race were cultured and monitored for the degree of ADM by microscopic duct counts over a 6-day period. The kinetic data for each of the 41 donors were modeled to a sigmoidal Emax model and the results of the individual plots are shown. Observed values are depicted as individual points whereas lines represent the corresponding predicted values to the Emax model.

**Supplemental Figure 6. Association between tumor stage, grade and subtype classification.** Tumor stage and grade distribution of PDAC samples from GSE183795 is shown along with the subtype classification of the normal adjacent to tumor (NAT samples). There is no significant difference in stage or grade distribution between the ERT and C/B groups (Pearson’s Chi-squared test, P>0.05). The observed variations are minor and likely reflect random variation rather than systematic differences.

**Supplemental Figure 7. Heatmap of gene expression from 281 normal pancreas.** Gene expression data for 281 normal pancreas tissues were mined from the GTEx (dbGaP) database; spectral clustering analysis was used to assign the data to subtypes Group 1 (ERT) and Group 2 (C/B) subtypes. The heatmap was generated for the assigned Group (horizontal) versus Basal (Moffitt), Classical (Moffitt) and Exocrine (Collisson) subtypes.

**Supplemental Figure 8. Activation of PI3K-AKT and Rho GTPase pathways in Group 2 normal pancreas.** Gene expression data of 281 (A,B) or 267 (C,D) normal pancreata from the dbGaP database was mined. The mean expression of the PI3K-AKT (A,C) or Rho GTPase (B,D) gene sets were stratified by Group (A,B) or by self-reported race (C,D). Two-tailed Mann-Whitney U-test.

**Supplemental Figure 9. Immune components are increased in C/B subtype of GTEx cohort.** Heatmap of the cytokine, chemokine and cytokine receptors gene expression in 281 samples of normal pancreases from the GTEx cohort as stratified by subtype ERT and C/B.

**Supplemental Figure 10. Increased expression of acinar transcription factors in Group 1 of NAT from independent cohort.** Gene expression data from the NAT from PDAC patients (GSE183795) was determined using cDNA arrays. The expression of three different acinar specific transcription factors (A) PTF1A, (B) BHLHA15 and (C) RBPJL are shown. Mean ± SD. Two-tailed Mann-Whitney U-test.

**Description of Supplemental Tables**

Supplemental Table 1. Donor Demographics

Supplemental Table 2. Gene signature for ADMI Up and ADMI Down

Supplemental Table 3. Results of spectral clustering

Supplemental Table 4. Results of Ancestry, Subtype and ADM Indices

Supplemental Table 5. Twenty housekeeping genes for Figure 5D

Supplemental Table 6. PI3K-AKT and Rho GTPase gene set used in Figure 5

**Supplemental References**

1. Jiang J, Hakimjavadi, H., Bray, J.K., Gosling, A., daSilva, L., Bulut, G., Perkins, C., Ali, J., Setiawan, V.W., Campbell-Thompson, M., Chamala, S., Schmittgen, T.D. . Transcriptional profile of human pancreatic acinar ductal metaplasia. Gastro Hep Advances **2023**;2:532-43

2. Da Silva L, Bray JK, Bulut G, Jiang J, Schmittgen TD. Method for improved integrity of RNA isolated from Matrigel cultures. MethodsX **2020**;7:100966

3. Ewels PA, Peltzer A, Fillinger S, Patel H, Alneberg J, Wilm A*, et al.* The nf-core framework for community-curated bioinformatics pipelines. Nat Biotechnol **2020**;38:276-8

4. Leek JT, Johnson WE, Parker HS, Jaffe AE, Storey JD. The sva package for removing batch effects and other unwanted variation in high-throughput experiments. Bioinformatics **2012**;28:882-3

5. Chen Y, Chen L, Lun ATL, Baldoni PL, Smyth GK. edgeR v4: powerful differential analysis of sequencing data with expanded functionality and improved support for small counts and larger datasets. Nucleic Acids Res **2025**;53

6. John CR, Watson D, Barnes MR, Pitzalis C, Lewis MJ. Spectrum: fast density-aware spectral clustering for single and multi-omic data. Bioinformatics **2020**;36:1159-66

7. Belleau P, Deschenes A, Chambwe N, Tuveson DA, Krasnitz A. Genetic Ancestry Inference from Cancer-Derived Molecular Data across Genomic and Transcriptomic Platforms. Cancer research **2023**;83:49-58

8. McKenna A, Hanna M, Banks E, Sivachenko A, Cibulskis K, Kernytsky A*, et al.* The Genome Analysis Toolkit: a MapReduce framework for analyzing next-generation DNA sequencing data. Genome Res **2010**;20:1297-303

9. Shen R, Seshan VE. FACETS: allele-specific copy number and clonal heterogeneity analysis tool for high-throughput DNA sequencing. Nucleic Acids Res **2016**;44:e131

10. Alexander DH, Lange K. Enhancements to the ADMIXTURE algorithm for individual ancestry estimation. BMC Bioinformatics **2011**;12:246
