# Supplementary material for: Transcriptomic Analyses of Normal Human Pancreata Reveal the Presence of Cancer Subtypes that Correlate with Acinar Ductal Metaplasia and Donor Ancestry
Source: Cancer Res Commun. 2026 Jan 21;6(1):165–77. doi: 10.1158/2767-9764.CRC-25-0411 (PMC12820465; doi:10.1158/2767-9764.CRC-25-0411)
Supplement: Supplementary Figure S1 — Figure S1. Principal component analysis (PCA) of bulk RNA-seq data from normal pancreatic acinar specimens. [file crc-25-0411_supplementary_figure_s1_suppsf1.pdf]

Supplemental Fig. 1

A

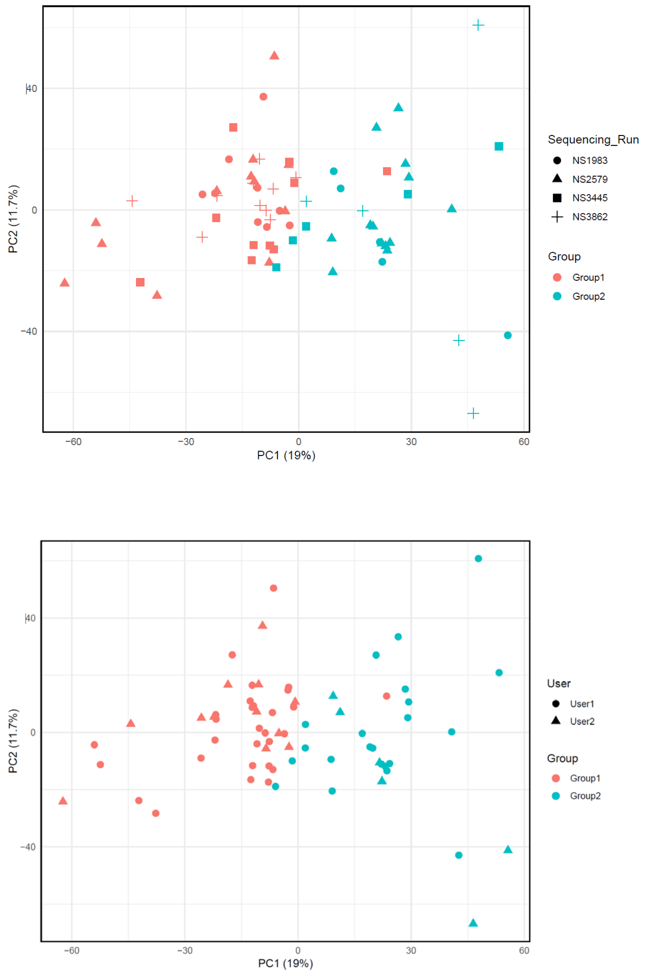

B

Supplemental Figure 1. Principal component analysis (PCA) of bulk RNA-seq data from normal pancreatic acinar specimens. Transcriptomic profiling was performed on 69 primary, normal pancreatic acinar samples. PCA was conducted using 14,346 filtered genes after batch correction, implemented with the PCAtools and ggplot2 packages in R. Samples are stratified by (A) Sequencing Run, and (B) Group. User to assess potential sources of technical variation.
