# Supplementary material for: Transcriptomic Analyses of Normal Human Pancreata Reveal the Presence of Cancer Subtypes that Correlate with Acinar Ductal Metaplasia and Donor Ancestry
Source: Cancer Res Commun. 2026 Jan 21;6(1):165–77. doi: 10.1158/2767-9764.CRC-25-0411 (PMC12820465; doi:10.1158/2767-9764.CRC-25-0411)
Supplement: Supplementary Figure S2 — Figure S2. GSEA of Group 1 and Group 2 data for 69 normal acinar specimens. [file crc-25-0411_supplementary_figure_s2_suppsf2.pdf]

Supplemental Fig. 2

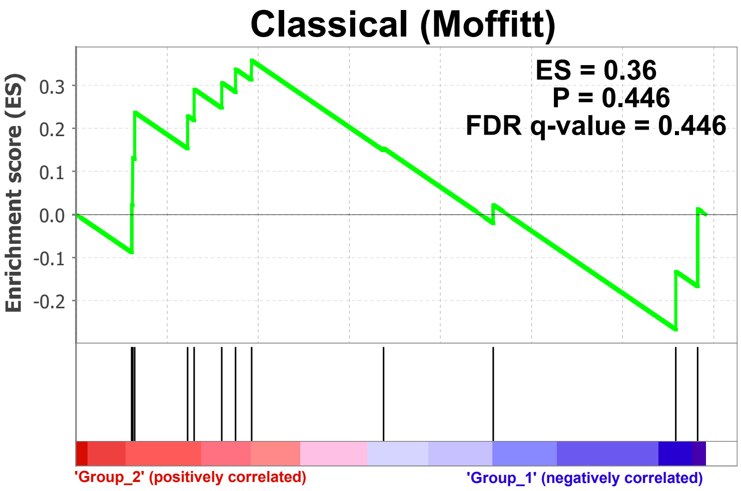

Supplemental Figure 2. GSEA of Group 1 and Group 2 data for 69 normal acinar specimens. GSEA was performed on the bulk RNA transcriptomic data from uncultured, normal human pancreatic acinar cells from 69 donors. Shown are the data from the Group 1 and Group 2 subtypes (ratio of Group 2/Group 1) using the classical (Moffitt) gene set.
