# Supplementary material for: Transcriptomic Analyses of Normal Human Pancreata Reveal the Presence of Cancer Subtypes that Correlate with Acinar Ductal Metaplasia and Donor Ancestry
Source: Cancer Res Commun. 2026 Jan 21;6(1):165–77. doi: 10.1158/2767-9764.CRC-25-0411 (PMC12820465; doi:10.1158/2767-9764.CRC-25-0411)
Supplement: Supplementary Figure S3 — Figure S3. Validation of ADMI on independent, data set. [file crc-25-0411_supplementary_figure_s3_suppsf3.pdf]

Supplemental Fig. 3

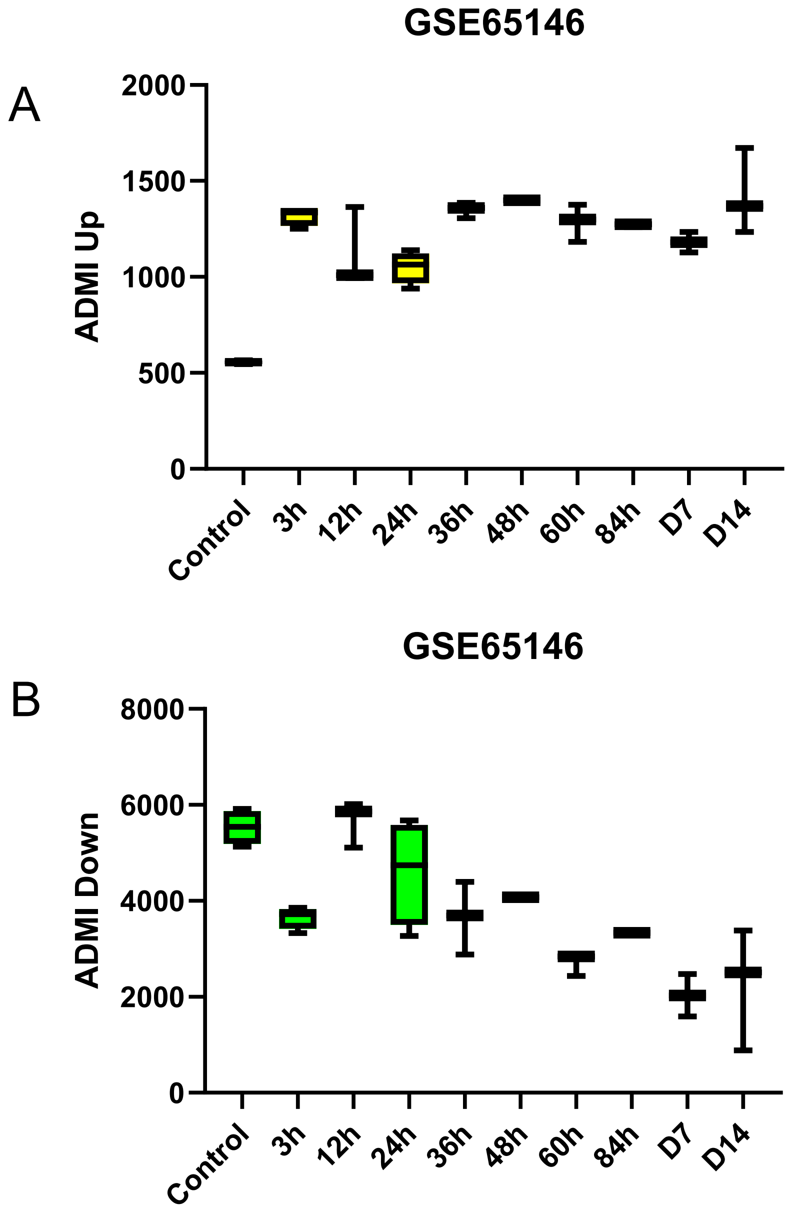

Supplemental Figure 3. Validation of ADMI on independent, data set. Data set GSE183795 was mined for pancreatitis mouse model injected with caerulein. Mice were sacrificed at various time post injections (x-axis) and the degree of pancreatitis. Shown are the ADMIUp (A) and ADMIDown (B) gene expression indices as a function of the degree of pancreatitis.
