# Supplementary material for: Transcriptomic Analyses of Normal Human Pancreata Reveal the Presence of Cancer Subtypes that Correlate with Acinar Ductal Metaplasia and Donor Ancestry
Source: Cancer Res Commun. 2026 Jan 21;6(1):165–77. doi: 10.1158/2767-9764.CRC-25-0411 (PMC12820465; doi:10.1158/2767-9764.CRC-25-0411)
Supplement: Supplementary Figure S4 — Figure S4. PCA based continental-level ancestry inference done with RAIDS software. [file crc-25-0411_supplementary_figure_s4_suppsf4.pdf]

Supplemental Fig. 4

PCA Profile 100620 SIRE: Hispanic GA: AMR

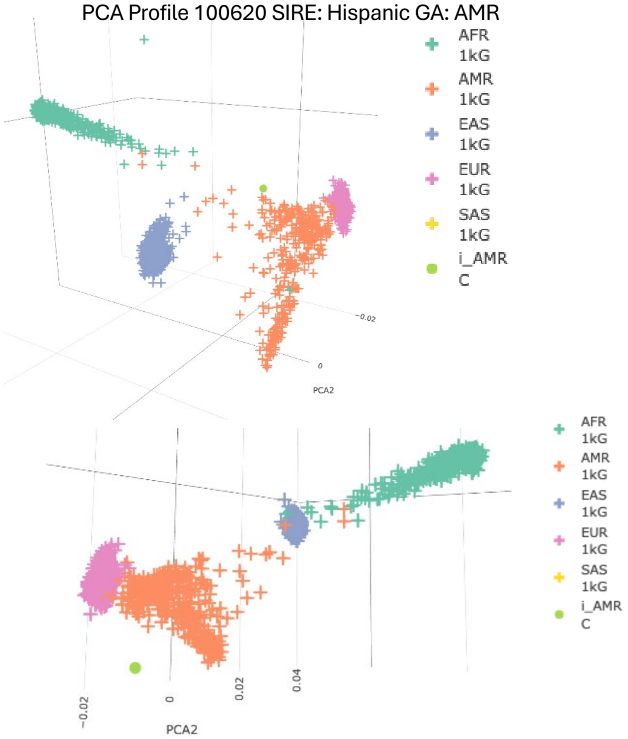

PCA Profile HP1132 SIRE: White GA: AMR

Supplemental Figure 4. PCA based continental-level ancestry inference done with RAIDS software. Examples of two Ameridigenous ancestry assigned patients from the current cohort (patients 1000620 and HP1132) using a PCA-based method. The star marks represent the 1000 Genomes cohort distribution according to their continental ancestry (AFR: green, AMR: orange, EAS: blue, EUR: pink, SAS: yellow). The round mark represents a patient of the current cohort.
