# Supplementary material for: Transcriptomic Analyses of Normal Human Pancreata Reveal the Presence of Cancer Subtypes that Correlate with Acinar Ductal Metaplasia and Donor Ancestry
Source: Cancer Res Commun. 2026 Jan 21;6(1):165–77. doi: 10.1158/2767-9764.CRC-25-0411 (PMC12820465; doi:10.1158/2767-9764.CRC-25-0411)
Supplement: Supplementary Figure S5 — Figure S5. ADM transdifferentiation kinetics as modeled to sigmoid Emax model. [file crc-25-0411_supplementary_figure_s5_suppsf5.pdf]

Observed vs Predicted ADM% by Sample ID and Race

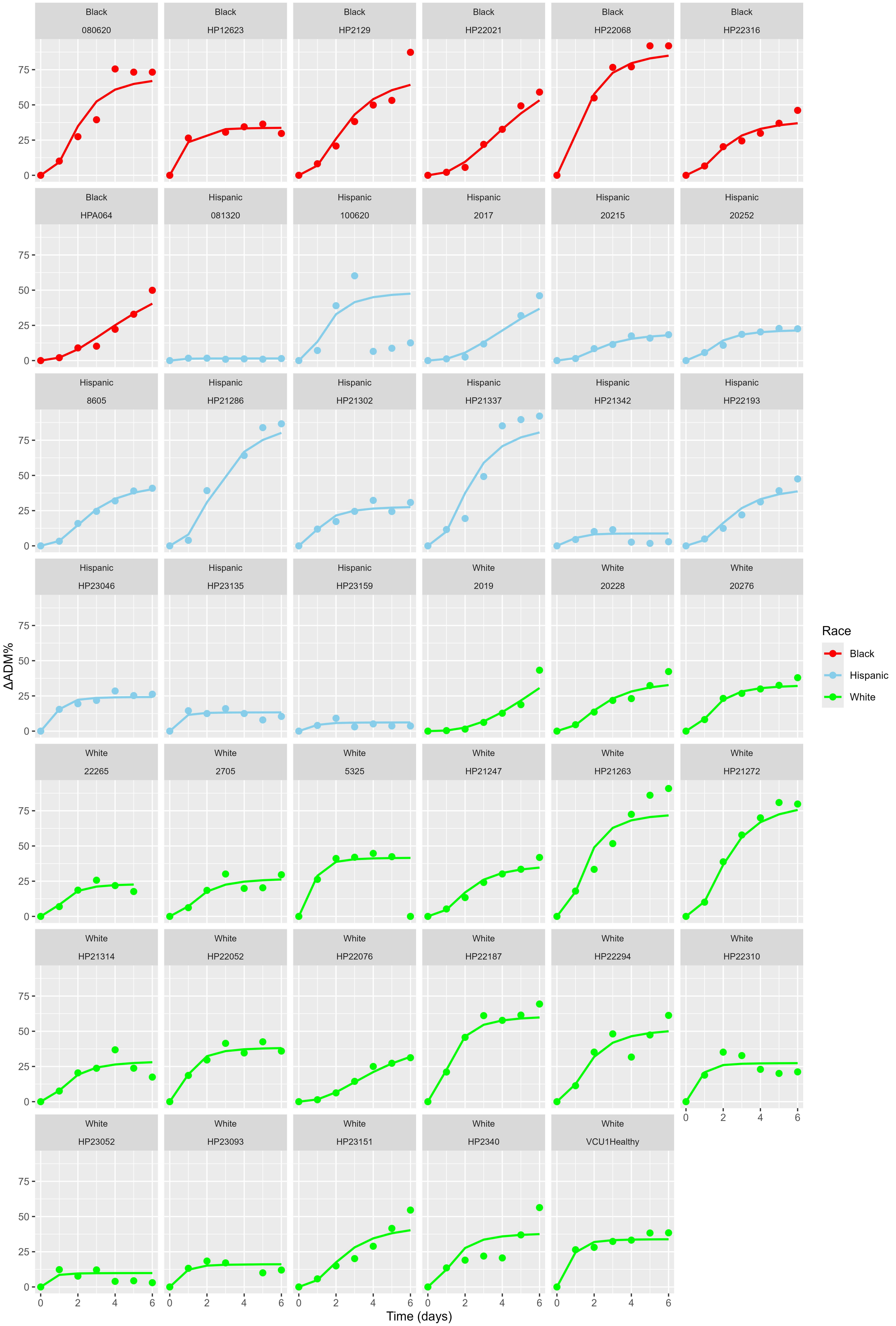

Supplemental Figure 5. ADM transdifferentiation kinetics as modeled to sigmoid Emax model. Pancreatic acinar cells from 41 deceased organ donors of differing self-identified race were cultured and monitored for the degree of ADM by microscopic duct counts over a 6-day period. The kinetic data for each of the 41 donors were modeled to a sigmoidal Emax model and the results of the individual plots are shown. Observed values are depicted as individual points whereas lines represent the corresponding predicted values to the Emax model.
