# Supplementary material for: Transcriptomic Analyses of Normal Human Pancreata Reveal the Presence of Cancer Subtypes that Correlate with Acinar Ductal Metaplasia and Donor Ancestry
Source: Cancer Res Commun. 2026 Jan 21;6(1):165–77. doi: 10.1158/2767-9764.CRC-25-0411 (PMC12820465; doi:10.1158/2767-9764.CRC-25-0411)
Supplement: Supplementary Figure S6 — Figure S6. Association between tumor stage, grade and subtype classification. [file crc-25-0411_supplementary_figure_s6_suppsf6.pdf]

Supplemental Fig. 6

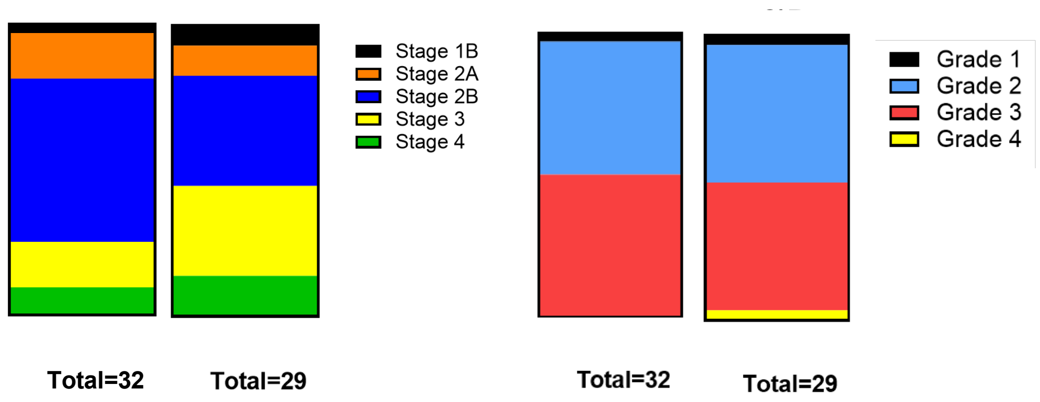

Supplemental Figure 6. Association between tumor stage, grade and subtype classification. Tumor stage and grade distribution of PDAC samples from GSE183795 is shown along with the subtype classification of the normal adjacent to tumor (NAT samples). There is no significant difference in stage or grade distribution between the ERT and C/B groups (Pearson's Chi-squared test, P>0.05). The observed variations are minor and likely reflect random variation rather than systematic differences.
