# Supplementary material for: Transcriptomic Analyses of Normal Human Pancreata Reveal the Presence of Cancer Subtypes that Correlate with Acinar Ductal Metaplasia and Donor Ancestry
Source: Cancer Res Commun. 2026 Jan 21;6(1):165–77. doi: 10.1158/2767-9764.CRC-25-0411 (PMC12820465; doi:10.1158/2767-9764.CRC-25-0411)
Supplement: Supplementary Figure S7 — Figure S7. Heatmap of gene expression from 281 normal pancreas. [file crc-25-0411_supplementary_figure_s7_suppsf7.pdf]

Supplemental Fig. 7

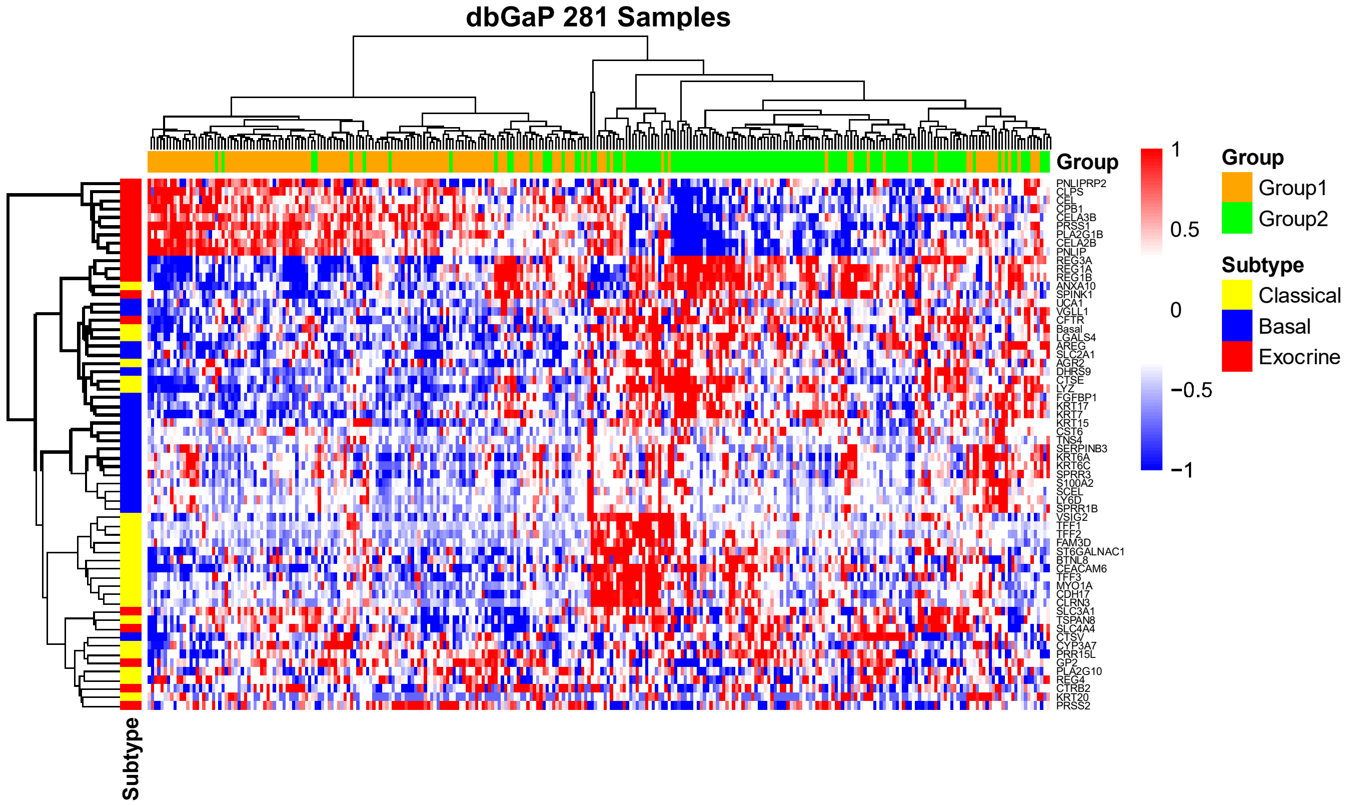

Supplemental Figure 7. Heatmap of gene expression from 281 normal pancreas. Gene expression data for 281 normal pancreas tissues were mined from the GTEx (dbGaP) database; spectral clustering analysis was used to assign the data to subtypes Group 1 (ERT) and Group 2 (C/B) subtypes. The heatmap was generated for the assigned Group (horizontal) versus Basal (Moffitt), Classical (Moffitt) and Exocrine (Collisson) subtypes.
