# Supplementary material for: Transcriptomic Analyses of Normal Human Pancreata Reveal the Presence of Cancer Subtypes that Correlate with Acinar Ductal Metaplasia and Donor Ancestry
Source: Cancer Res Commun. 2026 Jan 21;6(1):165–77. doi: 10.1158/2767-9764.CRC-25-0411 (PMC12820465; doi:10.1158/2767-9764.CRC-25-0411)
Supplement: Supplementary Figure S8 — Figure S8. Activation of PI3K-AKT and Rho GTPase pathways in Group 2 normal pancreas. [file crc-25-0411_supplementary_figure_s8_suppsf8.pdf]

Supplemental Fig. 8

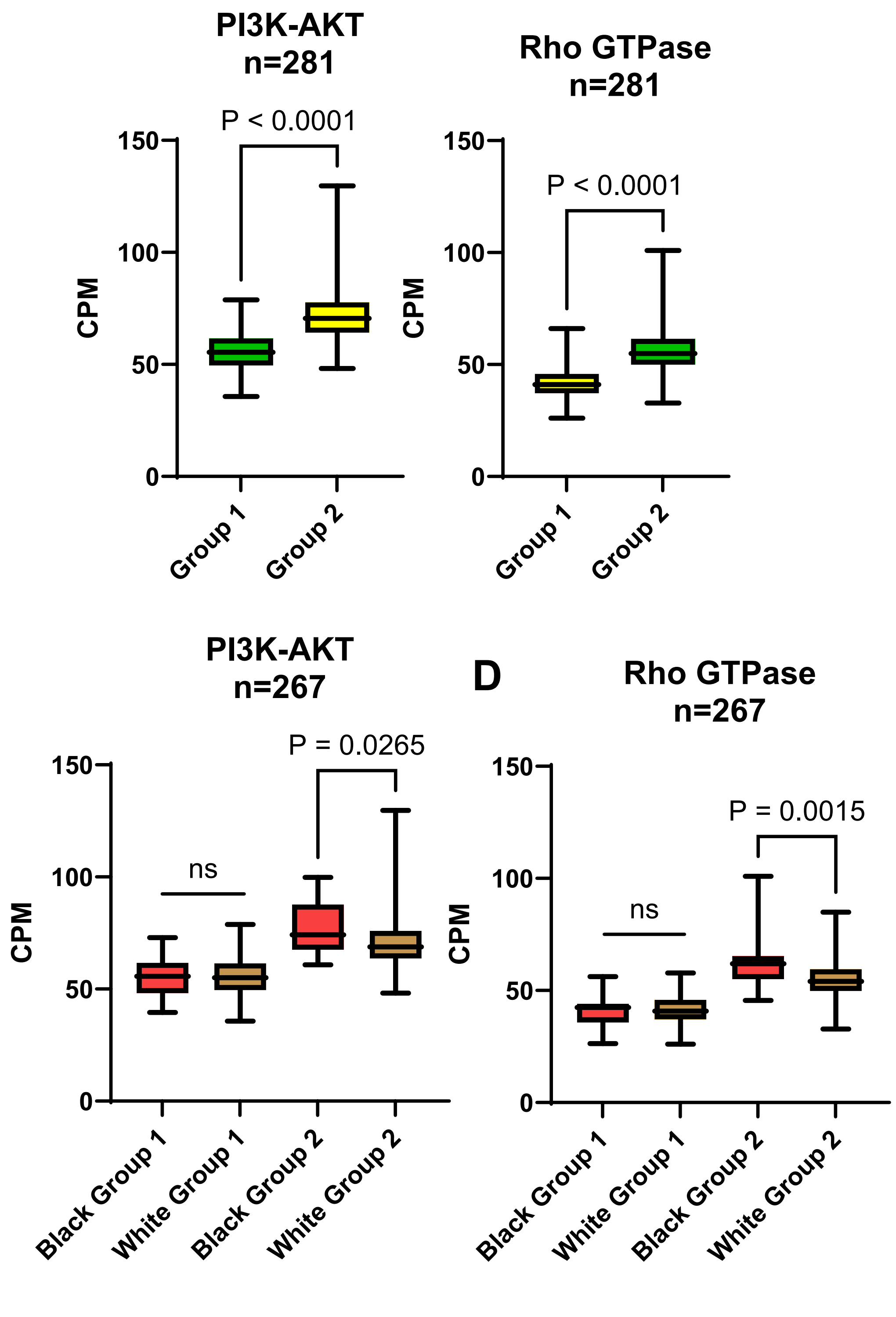

Supplemental Figure 8. Activation of PI3K-AKT and Rho GTPase pathways in Group 2 normal pancreas. Gene expression data of 281 (A,B) or 267 (C,D) normal pancreata from the dbGaP database was mined. The mean expression of the PI3K-AKT (A,C) or Rho GTPase (B,D) gene sets were stratified by Group (A,B) or by self-reported race (C,D). Two-tailed Mann-Whitney U-test.
