# Supplementary material for: Transcriptomic Analyses of Normal Human Pancreata Reveal the Presence of Cancer Subtypes that Correlate with Acinar Ductal Metaplasia and Donor Ancestry
Source: Cancer Res Commun. 2026 Jan 21;6(1):165–77. doi: 10.1158/2767-9764.CRC-25-0411 (PMC12820465; doi:10.1158/2767-9764.CRC-25-0411)
Supplement: Supplementary Figure S9 — Figure S9. Immune components are increased in C/B subtype of GTEx cohort. [file crc-25-0411_supplementary_figure_s9_suppfs9.pdf]

Supplemental Fig. 9

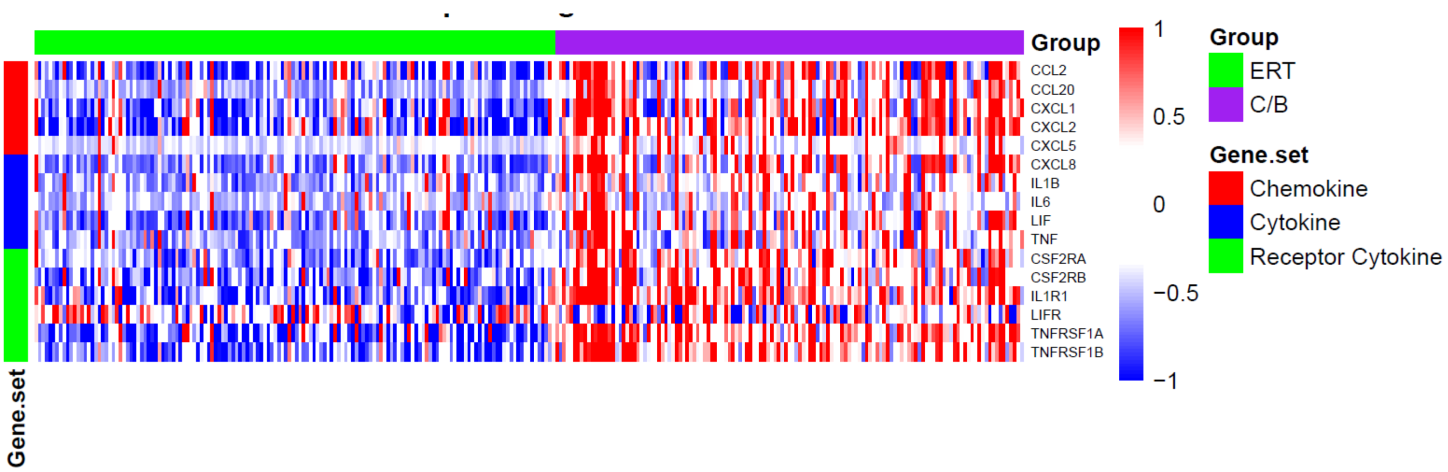

Supplemental Figure 9. Immune components are increased in C/B subtype of GTEx cohort. Heatmap of the cytokine, chemokine and cytokine receptors gene expression in 281 samples of normal pancreases from the GTEx cohort as stratified by subtype ERT and C/B.
