# Supplementary material for: Transcriptomic Analyses of Normal Human Pancreata Reveal the Presence of Cancer Subtypes that Correlate with Acinar Ductal Metaplasia and Donor Ancestry
Source: Cancer Res Commun. 2026 Jan 21;6(1):165–77. doi: 10.1158/2767-9764.CRC-25-0411 (PMC12820465; doi:10.1158/2767-9764.CRC-25-0411)
Supplement: Supplementary Figure S10 — Figure S10. Increased expression of acinar transcription factors in Group 1 of NAT from independent cohort. [file crc-25-0411_supplementary_figure_s10_suppsf10.pdf]

Supplemental Fig. 10

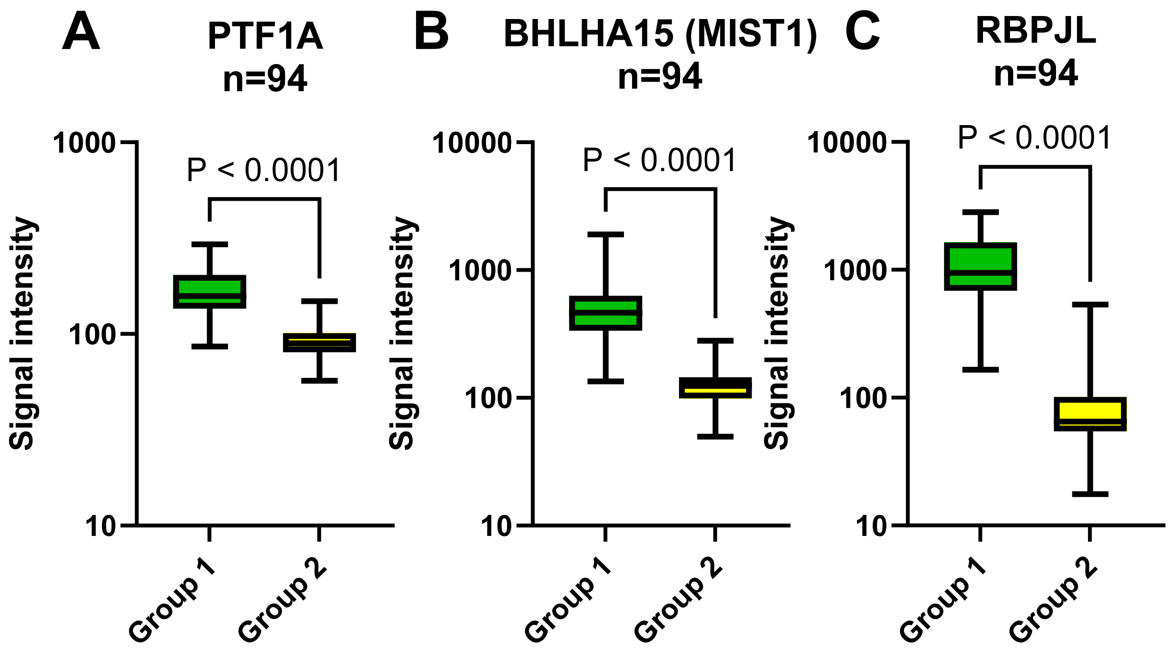

Supplemental Figure 10. Increased expression of acinar transcription factors in Group 1 of NAT from independent cohort. Gene expression data from the NAT from PDAC patients (GSE183795) was determined using cDNA arrays. The expression of three different acinar specific transcription factors (A) PTF1A, (B) BHLHA15 and (C) RBPJL are shown. Mean ± SD. Two-tailed Mann-Whitney U-test.
